# Experimental and Theoretical Studies on Acid Corrosion Inhibition of API 5L X70 Steel with Novel 1-*N*-α-d-Glucopyranosyl-1*H*-1,2,3-Triazole Xanthines

**DOI:** 10.3390/molecules28010460

**Published:** 2023-01-03

**Authors:** Alma Sánchez-Eleuterio, Carlos Mendoza-Merlos, Ricardo Corona Sánchez, Alejandra M. Navarrete-López, Anatolio Martínez Jiménez, Elsie Ramírez-Domínguez, Leticia Lomas Romero, Ricardo Orozco Cruz, Araceli Espinoza Vázquez, Guillermo E. Negrón-Silva

**Affiliations:** 1 Departamento de Ciencias Básicas, Universidad Autónoma Metropolitana, Av. San Pablo No. 180, Azcapotzalco, Ciudad de Mexico 02200, Mexico; 2 Departamento de Química, Universidad Autónoma Metropolitana, Av. San Rafael Atlixco No. 186, Ciudad de Mexico 09340, Mexico; 3 Instituto de Ingeniería, Universidad Veracruzana, Av. S. S. Juan Pablo II S/N, Boca del Río, Veracruz 94294, Mexico

**Keywords:** corrosion inhibition, steel, carbohydrates, xanthines, triazole, DFT

## Abstract

A series of novel 1-*N*-α-d-glucopyranosyl-1*H*-1,2,3-triazole xanthines was synthesized from azido sugars (glucose, galactose, and lactose) and propargyl xanthines (theophylline and theobromine) using a typical copper (I)-catalyzed azide–alkyne 1,3-dipolar cycloaddition. The corrosion inhibition activities of these new carbohydrate-xanthine compounds were evaluated by studying the corrosion of API 5 L X70 steel in a 1 M HCl medium. The results showed that, at 10 ppm, a 90% inhibition efficiency was reached by electrochemical impedance spectroscopy. The inhibitory efficiency of these molecules is explained by means of quantum chemical calculations of the protonated species with the solvent effect, which seems to better represent the actual situation of the experimental conditions. Some quantum chemical parameters were analyzed to characterize the inhibition performance of the tested molecules.

## 1. Introduction

Steel and its alloys are widely used to manufacture different equipment in various industries because of their high durability and exceptional mechanical properties; however, they are prone to mechanical and chemical damage. Steel corrosion remains one of the most significant problems for the industry, and this phenomenon has tremendous negative economic effects [1,2]. Among the different prevention and control methods to avoid this problem, the use of corrosion inhibitors is widespread [3,4]. Although many effective corrosion inhibitors have been reported, there is a renewed interest in the field of organic compounds due to the increasing demand for efficient, low-cost, and non-toxic corrosion inhibitors [5,6,7]. The efficiency of an organic compound as a corrosion inhibitor is closely associated with its chemical adsorption [8]. The adsorption of organic molecules at the metal surface creates a barrier between it and the electrolytic phase, thus slowing down the mechanism of corrosion by forming a protective layer on the metal surface [9].

The effectiveness of an organic corrosion inhibitor depends on several structural features, which include molecular geometry, steric factors, the presence of electron-donating/withdrawing functional groups, aromaticity, structural planarity, etc. [10]. Commonly, organic compounds that contain heteroatoms, such as nitrogen, oxygen, and sulfur, and those that contain unsaturated π-bonds, and conjugated aromatic rings are good candidates for testing as corrosion inhibitors [11,12,13]. The planarity of the orbital and the lonely electron pairs in the heteroatom are important features that determine the adsorption of these molecules on the metallic surface [14].

Different classes of organic compounds have been used as corrosion inhibitors; however, the investigation of natural organic compounds is particularly interesting because of their relatively low cost and because they are considered environmentally safe [15,16,17]. In this context, carbohydrates [18,19,20,21,22,23] and xanthine derivatives such as theophylline [24,25], theobromine [26], and caffeine [14,18,27,28,29,30,31,32,33] have been used as corrosion inhibitors of metals.

Carbohydrates are the most abundant eco-friendly materials in natural resources and are very useful biomaterials for several applications. It has been reported that some carbohydrate derivatives could act as efficient, eco-friendly inhibitors [19]. Moreover, caffeine is a naturally occurring molecule and a non-toxic, environmentally friendly substance found in several foods or drug formulations, whose use as a corrosion inhibitor has been evaluated for a series of metals and alloys [28,29,30,31,32,33]. A few reports have disclosed the capacity of theophylline or theobromine derivatives as organic corrosion inhibitors in metals under an acidic medium. For instance, a theophylline derivative bearing a thiobenzimidazole moiety showed excellent inhibition efficiency in preventing the corrosion of aluminum in a 1 M HCl medium [34]. The inhibitory behavior of a related theophylline derivative was tested as a copper corrosion inhibitor in a 1 M HNO_3_ solution [35]. Additionally, theophylline has been studied as a corrosion inhibitor using API 5L X70 steel and has proved effective at low concentrations [25]. The ability of theobromine to inhibit mild steel and aluminum corrosion processes in an acidic medium has also been confirmed [26].

Moreover, we have previously demonstrated that some 1,2,3-triazole derivatives can also be considered effective corrosion inhibitors for carbon steels in an acidic medium [36,37,38,39,40,41]. Therefore, this study aims to investigate the inhibitive properties of novel carbohydrate-xanthine conjugates linked through a 1,2,3-triazole ring on the corrosion of the steel API 5L X70 in 1 M HCl, using both experimental (electrochemical impedance spectroscopy (EIS)), and theoretical (density functional theory (DFT)) approaches to study if a synergistic inhibitory corrosion effect could be achieved.

## 2. Results and Discussion

### 2.1. Synthesis

**Scheme 1:**
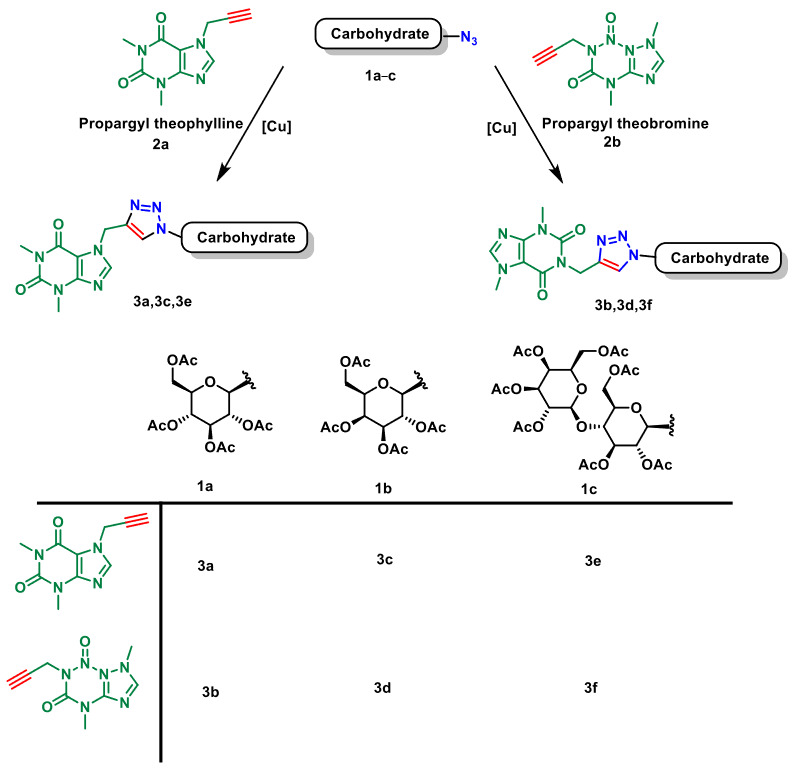
Synthesis of carbohydrate-xanthine conjugates **3a**–**f**.

The synthetic strategy adopted for the synthesis of carbohydrate-xanthine conjugates is shown in Scheme 1. The precursor azido sugars **1a**–**c** and propargyl xanthines **2a**,**b** were synthesized according to the previously reported procedures [42]. Then, the desired carbohydrate-xanthine conjugates **3a**–**f** were synthesized using a typical copper (I)-catalyzed azide–alkyne 1,3-dipolar cycloaddition. After chromatographic purification, the corresponding triazoles were obtained in good yields and were characterized by 1 H NMR, ^13^C NMR, and HRMS.

### 2.2. Corrosion Inhibition Studies

#### 2.2.1. Open Potential Circuit of Carbohydrate-Xanthine Conjugates

**Figure 1:**
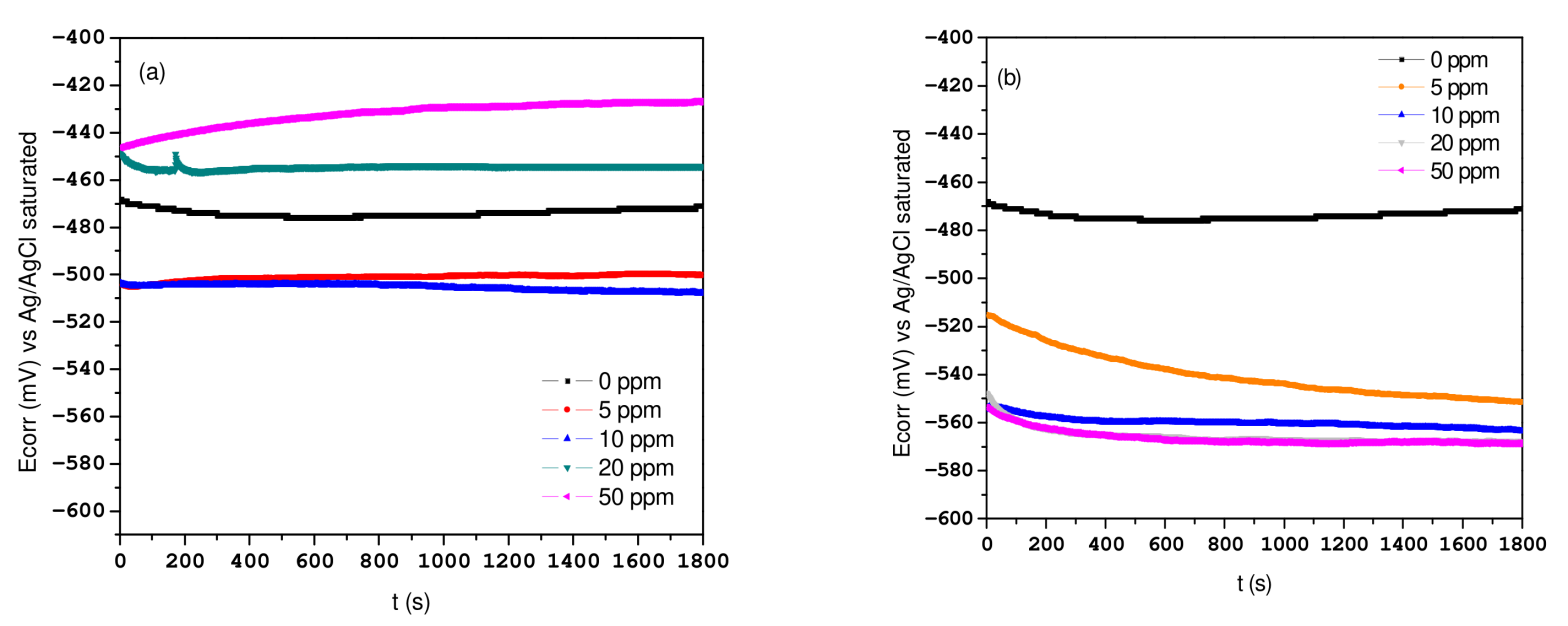

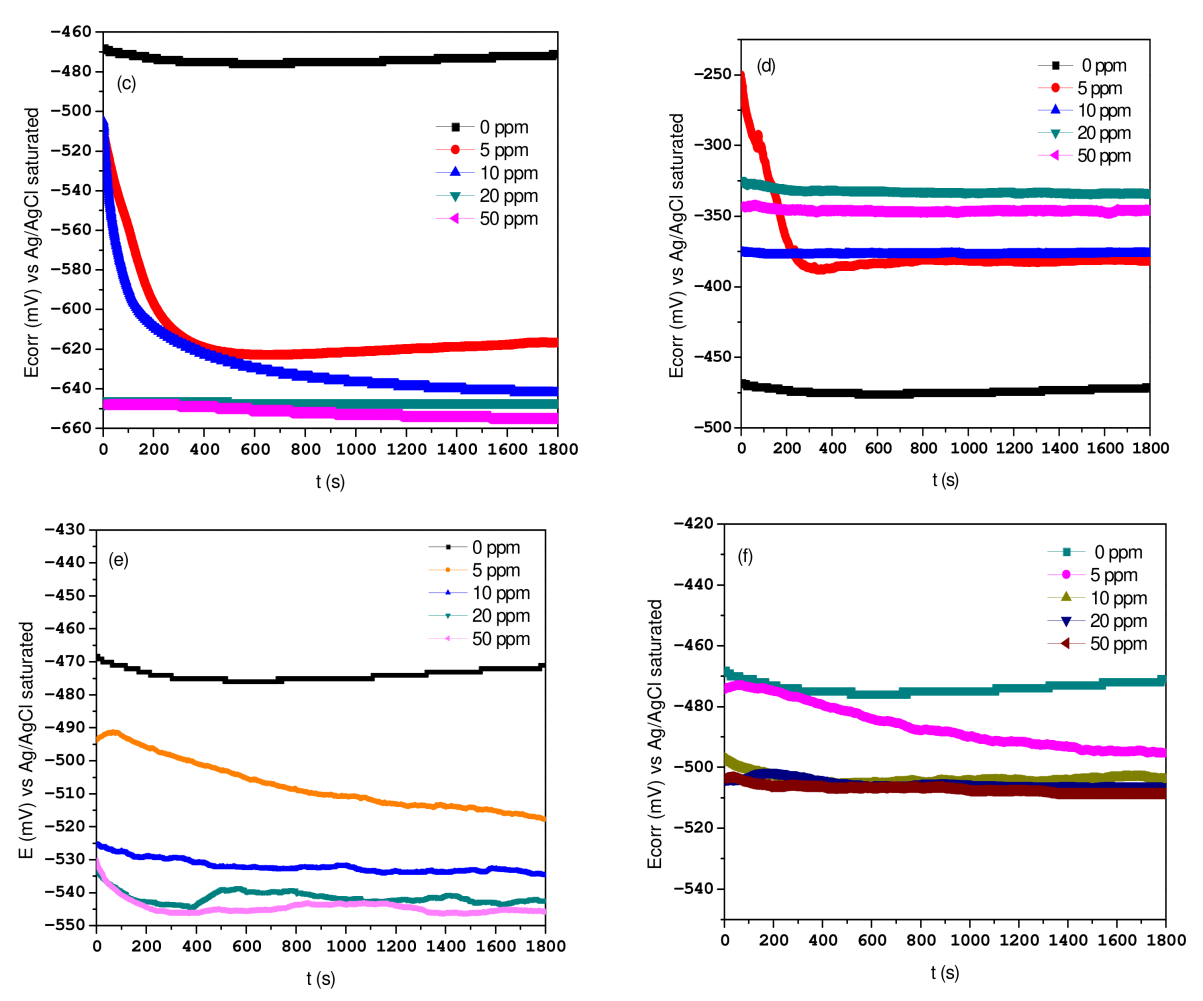
OCP plots at different concentrations of (**a**) compound **3a**, (**b**) compound **3b**, (**c**) compound **3c**, (**d**) compound **3d**, (**e**) compound **3e**, and (**f**) compound **3f** immersed in HCl 1 M.

The OCP at the different inhibitors is monitored until a steady state is obtained at the surface of API 5L X70. In general, as shown in Figure 1, the Ecorr is observed to remain stable from 1000 s for carbohydrate xanthine conjugates.

#### 2.2.2. Effect of Concentration by EIS

**Figure 2:**
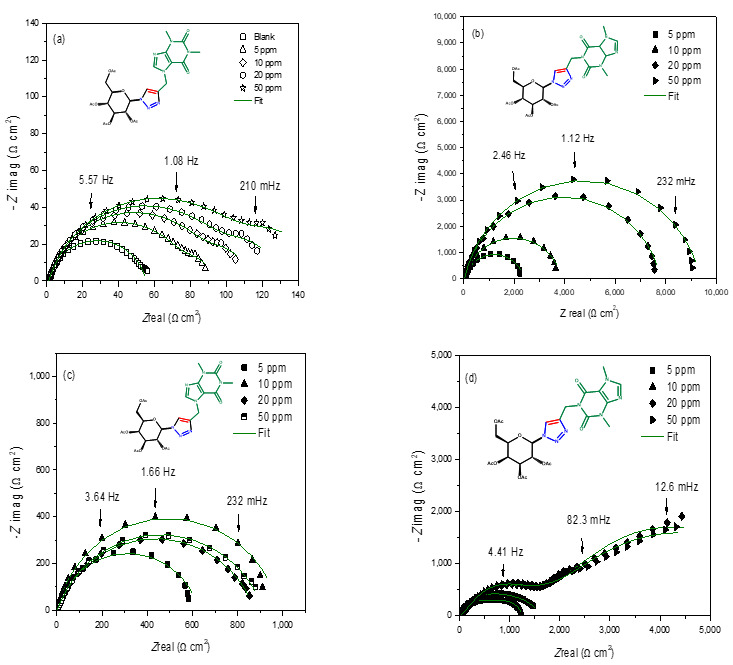

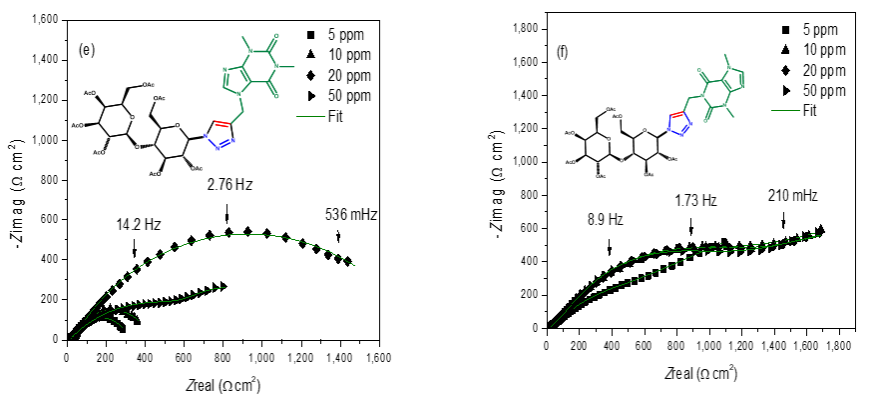
Nyquist diagram at different inhibitor concentrations (**a**) compound **3a**, (**b**) Compound **3b,** (**c**) compound **3c,** (**d**) Compound **3d,** (**e**) compound **3e** and (**f**) compound **3f** in API 5L X70 steel immerse in HCl 1 M.

The Nyquist diagrams at different concentrations and six organic compounds are shown in Figure 2. In Figure 2a, corresponding to inhibitor **3a**, it is noticeable that the maximum semicircle diameter was reached up to a concentration of 50 ppm of the inhibitor under static conditions, which is attributed to the fact that it was at a greater concentration with Zreal 140 Ω cm^2^. However, when inhibitor **3b** was evaluated under the same conditions, a better adsorption of the inhibitor was observed, as the Zreal value was higher than that of compound **3a**, which can be explained because the substituent, when changing its position, has a better interaction with the metal surface. For inhibitor **3b,** the Nyquist plot was controlled by charge transfer resistance (Figure 2b) [36].

Alternatively, when the inhibitor **3c** was evaluated (Figure 2c), the Nyquist plot showed an increase in the semicircle diameter, when the 10 ppm inhibitor concentration was increased, which remained practically constant up to 50 ppm. This observation was attributed to the fact that the inhibitor reached its maximum coverage degree to protect the metal surface. In the case of inhibitor **3d**, two constants were noticeable, attributed to charge transference resistance and film organic resistance (Figure 2d) [37].

Then, the inhibitor **3e** showed similar results to the inhibitor **3c** and the best concentration was 20 ppm with Zreal ~1800 Ω cm^2^. Finally, the inhibitor **3e** (Figure 2e) showed similar behavior at low concentrations.

**Figure 3:**
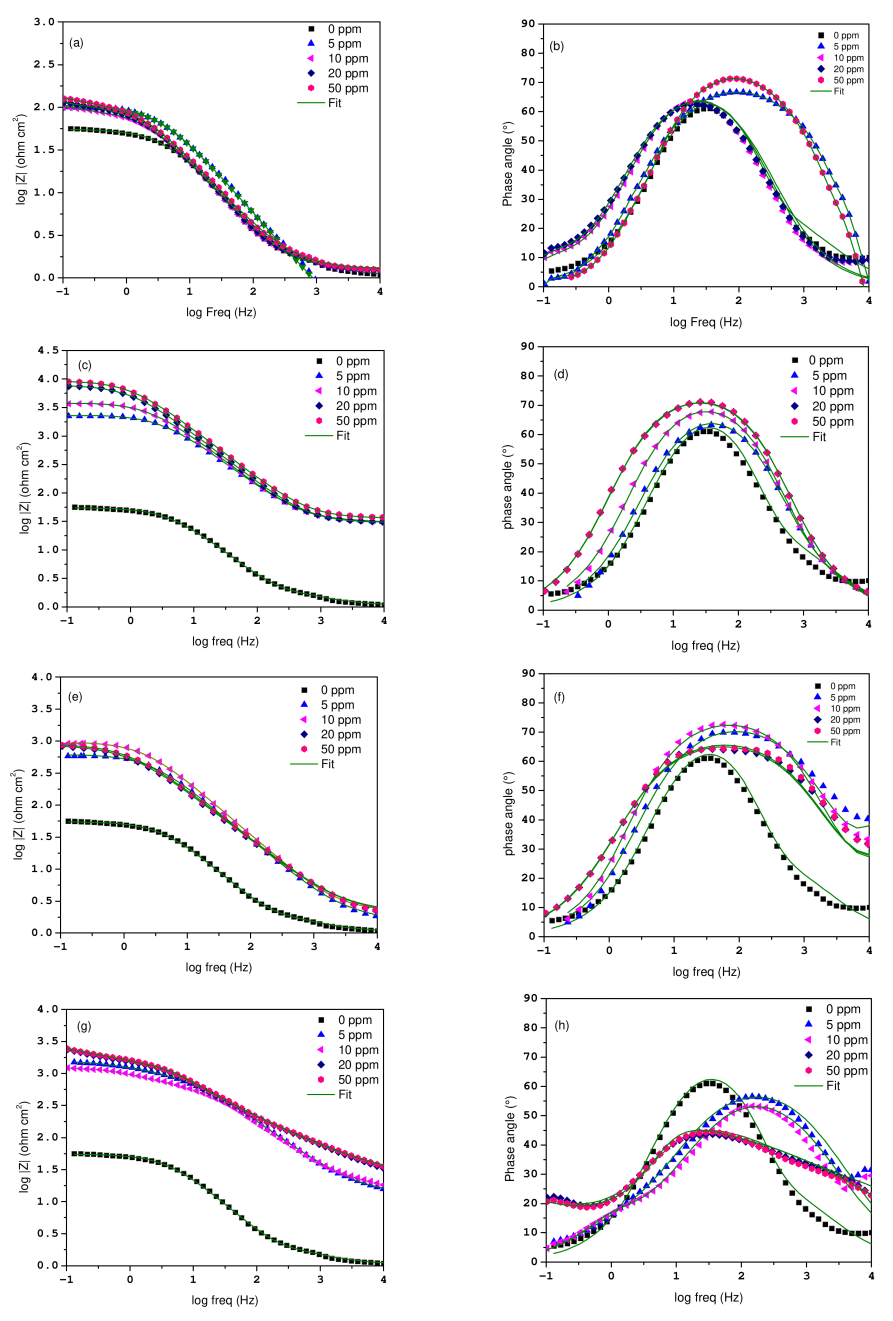

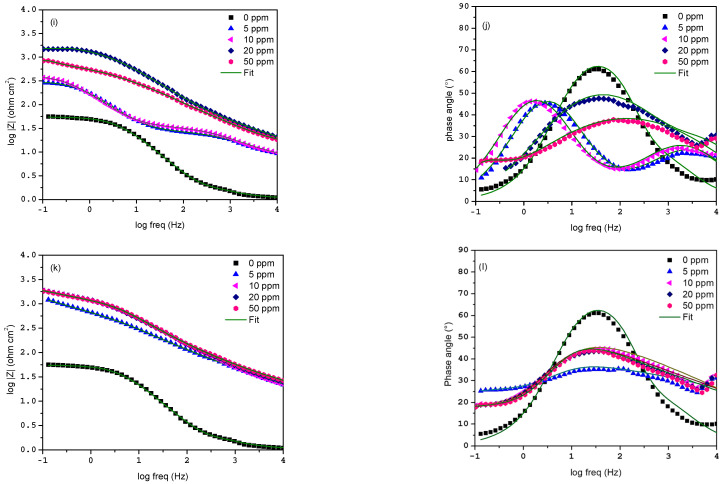
Bode diagram at different inhibitor concentrations (**a**,**b**) compound **3a**, (**c**,**d**) compound **3b**, (**e**,**f**) compound **3c**, (**g**,**h**) compound **3d**, (**i**,**j**) compound **3e**, and (**k**,**l**) compound **3f** in API 5L X70 steel immersed in HCl 1 M.

The Bode plot results are shown in Figure 3, and it can be seen that the Bode impedance modulus increases with the addition of corrosion inhibitors **3a**–**3f**. While, with increasing the concentration of 1-*N*-α-d-glucopyranosyl-1*H*-1,2,3-triazole xanthines, the frequency ranges with the maximum phase angle increase, indicating effective adsorption of inhibitor molecules on the metal surface. For the Bode plot, it is also observed that the phase angle broadens in the inhibitor’s presence or shows the two contributions in the intermediate frequency range, which implies the presence of two time constants.

**Figure 4:**
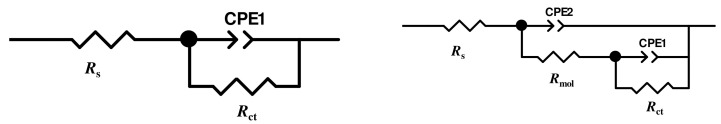
Equivalent electrical circuits.

In Figure 4, the different equivalent circuits used for the impedance results simulation are shown, where $R_{s}$ is the solution resistance, $R_{ct}$ is the charge transfer resistance, $R_{F}$ is the inhibitor film resistance, and Q is the phase constant element.

The double layer capacitance $\left(C_{dl}\right)$ was calculated by [38]:

(1) $$C_{dl}=Y_{0}^{\frac{1}{n}}{\left[\left(\frac{1}{R_{S}}\right)+\left(\frac{1}{R_{ct}}\right)\right]}^{\frac{n-1}{n}}$$

where $Y_{0}$ is a proportion factor and n is the phase change, which can take values of 0 for a resistance, 1 for a capacitance and −1 for an inductance.

The inhibition efficiency (*IE*(%)) was calculated by [39]:

(2) $$IE\left(\%\right)=\frac{\left(\frac{1}{Rp}\right)inhibitor-\left(\frac{1}{R_{p}}\right)blank}{\left(\frac{1}{R_{p}}\right)inhibitor}\times 100$$

where: *R_p_ blank*: Polarization resistance without inhibitor and *R_p_ inhibitor*.

The electrochemical parameters for the inhibitors are listed in Table 1. The *R_ct_* value increases in order as the inhibitor concentration increases in all cases. On the other hand, the corrosion inhibitor protection of the surface leads the *C_dl_* values to decrease. This observation was attributed to the variation in *R_ct_* and *C_dl_* values, which can be correlated with the gradual movement of the water molecules over the surface of the electrode in the presence of triazole xanthines, leading to a decrease in active sites and delaying the corrosion process [40].

**Figure 5:**
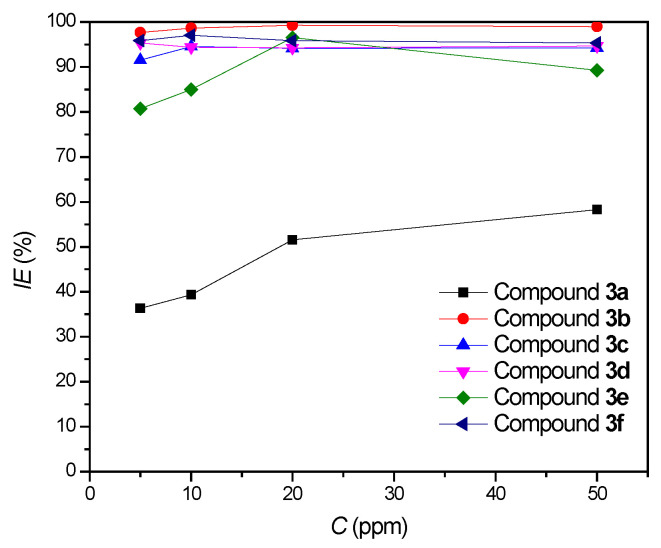
Variation of inhibition efficiency by xanthine triazole for API 5L X70 immersed in HCl 1 M.

Figure 5 shows the behavior of the inhibitors studied, where the inhibition efficiency values at different concentrations are shown, observing that compounds **3b**, **3c**, **3d**, and **3g** from 5 ppm have >90% protection against corrosion and fulfil their efficiency according to the reference standard NRF-005-2009.

Interestingly, compound **3a** shows corrosion protection attributed to the protective film, which is more easily desorbed from the metal surface.

Finally, compound **3e** shows that the best corrosion protection was at 20 ppm with 96.5% *IE*. Overall, it can be attributed to the fact that the fragment responsible for such high inhibition efficiency is the xanthine part, and that is corroborated by the theoretical simulation part.

#### 2.2.3. Xanthine Triazole Concentration by CP

**Figure 6:**
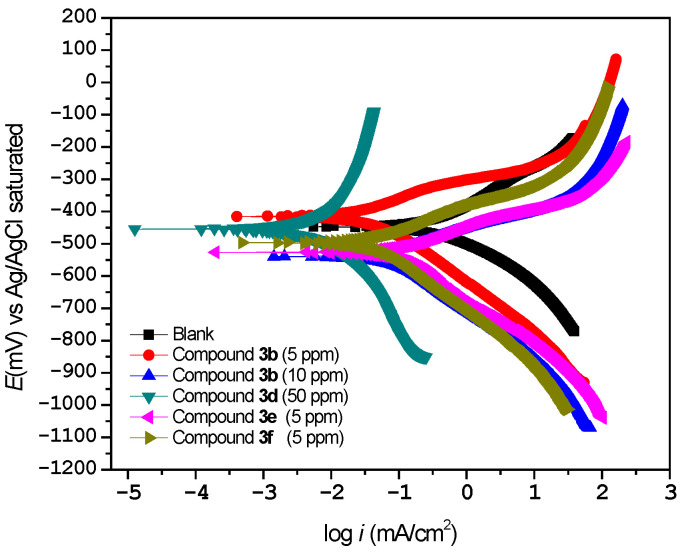
Polarization curves of API 5L X65 steel at different concentrations of inhibitor in saline medium.

The Tafel polarization curves were evaluated for the API 5L X70 steel in contact with HCl 1 M in the absence and presence of different xanthine triazoles, in order to obtain comparison parameters between the blank and the inhibitor, are shown in Figure 6.

The electrochemical parameters such as corrosion potential (*E*_corr_), current density (i*_corr_*), cathodic Tafel slope (b_c_), and anodic Tafel slope (b_a_) are calculated by the Tafel linear extrapolation method according to the CP curves. These electrochemical parameters are listed in Table 2, and the inhibition efficiency (η (%)) by this technique was calculated by:

(3) $$\eta \left(\%\right)=\frac{i_{corr}{}^{blank}-{\;i}_{corr}{}^{inh}}{i_{corr}{}^{blank}}\times 100$$

where i*_corr_^blank^* and i*_corr_^inh^* are the current density of corrosion in the absence and presence of inhibitor respectively.

Table 2 shows the electrochemical parameters obtained from the extrapolation of the anodic and cathodic polarization curves for the different concentrations of xanthines in 1 M hydrochloric acid. It is observed that the i*_corr_* values decrease in the presence of the inhibitor, which is attributed to the fact that it is retarding the dissolution process of the metal. On the other hand, the *E*_corr_ shows a different value that is more than 85 mV, this value suggests that the inhibitors are of the mixed type. Finally, the efficiencies calculated by this technique are similar and show the same behavior as those obtained by the electrochemical impedance spectroscopy technique.

#### 2.2.4. Adsorption Process

The adsorption isotherms can provide important information about the interaction of the inhibitor with the metallic surface. The types of interactions that can be described are physisorption and chemisorption processes. The former is held among the positive active centers of the electrons of the benzene rings with the metallic surface, while the latter is due to the formation of the coordination bonds between the molecules of the inhibitor and the d orbitals of the atom of steel over the steel surface. The most common model that describes the behavior of inhibitors is the Langmuir model, which mentions that a monolayer of organic inhibitor can be adsorbed on the metallic surface [41]:

(4) $$\frac{C}{\theta }=\frac{1}{k_{ads}}+C$$

where *C* is the inhibitor concentration in mol/L, *θ* is the coverage degree, and *k_ads_* is the equilibrium constant.

After analyzing the best isotherm that describes the behavior of the inhibitors, the Gibbs standard energy of adsorption was calculated [43]:

(5) $$\Delta G{{}^{\circ}}_{ads}=-55.5RTlnk_{ads}$$

where *R* is the constant of ideal gases, *T* is the absolute temperature, and *k_ads_* is the equilibrium constant.

**Figure 7:**
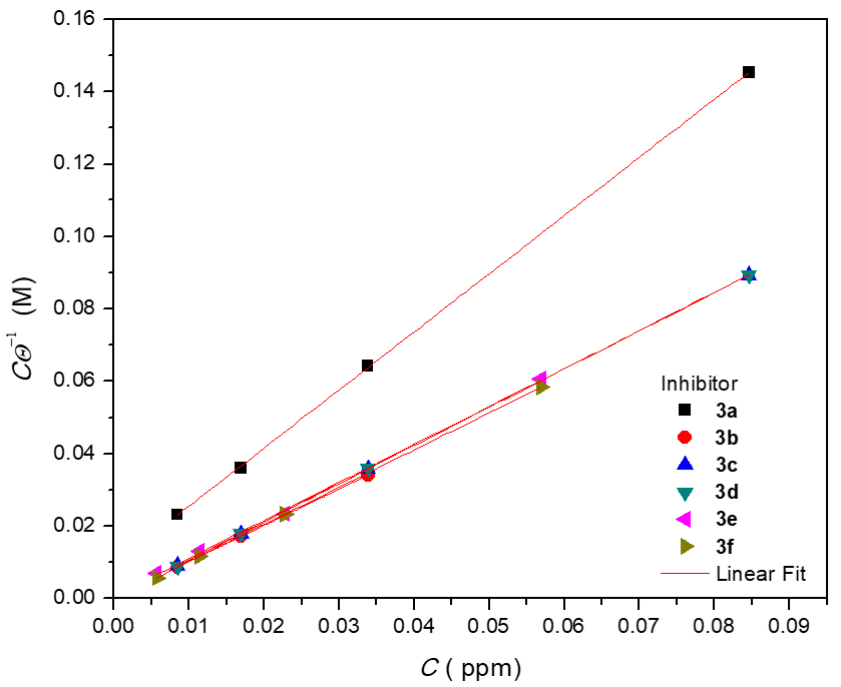
Adsorption isotherm of carbohydrate-xanthine conjugates.

As shown in Figure 7, it was found that *C*/*θ* vs. *C* was a straight line, with linear regression coefficients close to 1. According to these results, the important mechanism of inhibitor adsorption was obtained and is shown in Table 2.

In Table 3, the thermodynamic parameters are shown. It is important to mention that the Δ*G*°*_ads_* is lower than −20 kJ/mol, which, according to the literature, implies a physisorption process for inhibitor **3a**. In this case, chemisorption is greater than or equal to −40 kJ/mol, with combined having a value between −20 and −40 kJ/mol [44]. The results in Table 2 show that the adsorption process of the **3b**–**3f** inhibitor resulted from a combination of physisorption and chemisorption.

#### 2.2.5. Mechanism of Corrosion Inhibition by the New Carbohydrate-Xanthine

The poor inhibition shown in the **3a** organic inhibitor could be attributed to the spatial arrangement of its substituents, which leads to a weak electronic interaction by the OAc substituents in the pyranoic ring on the metal surface, adding to the poor electronic nature compared to **3e**.

**Figure 8:**
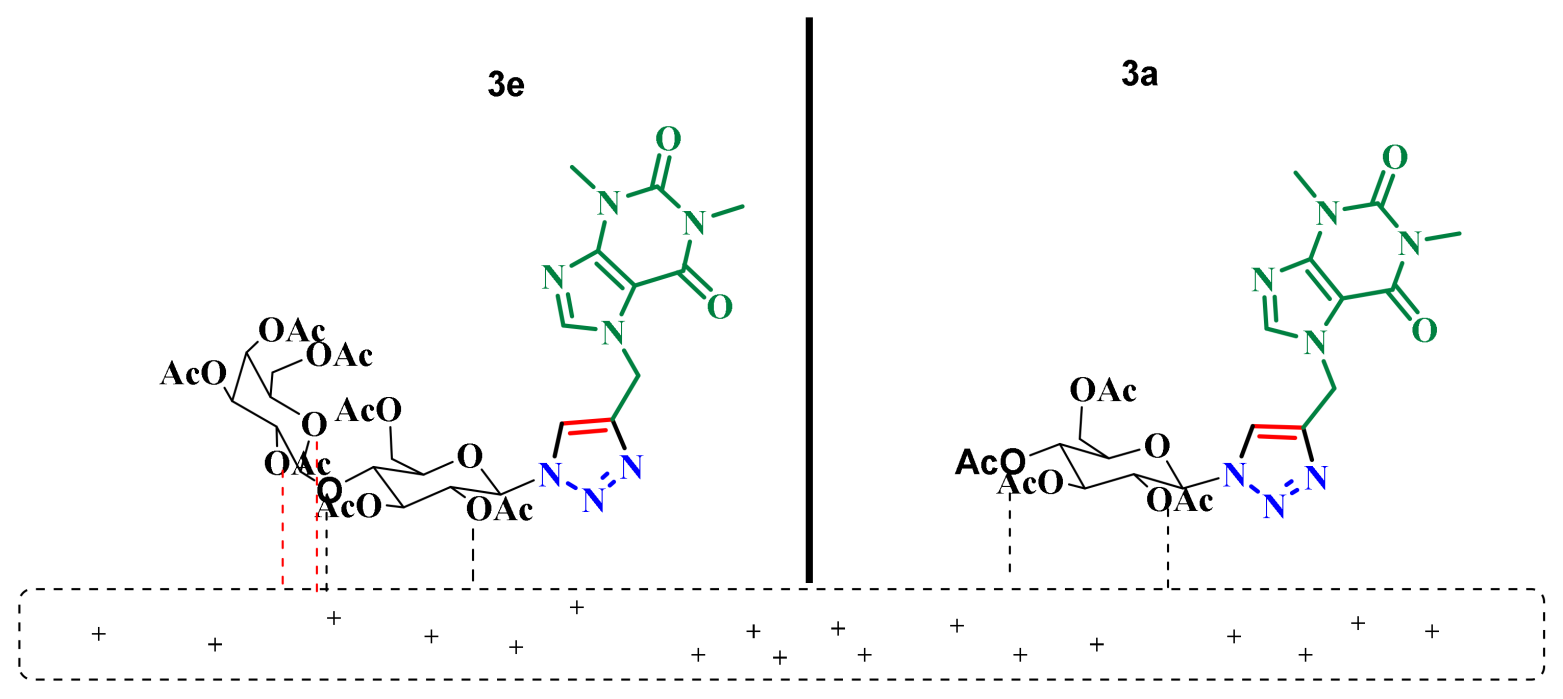
Comparison of inhibitor adsorption by compound **3e** and **3a** in metal surface.

With this idea in mind, we could suggest that while in **3e** there is a strong electronic contribution from the OAc substituents of the pyranoic rings present in lactose on the metallic surface, this electronic contribution in **3a** is minimal, which could explain why **3e** shows much higher corrosion inhibition efficiency than **3a** (Figure 8). 

In the same way, we propose that another of the factors that reduce the electronic contribution and therefore a decrease in the inhibition of corrosion for **3a** could be due to the stereochemistry shown in these compounds.

**Figure 9:**
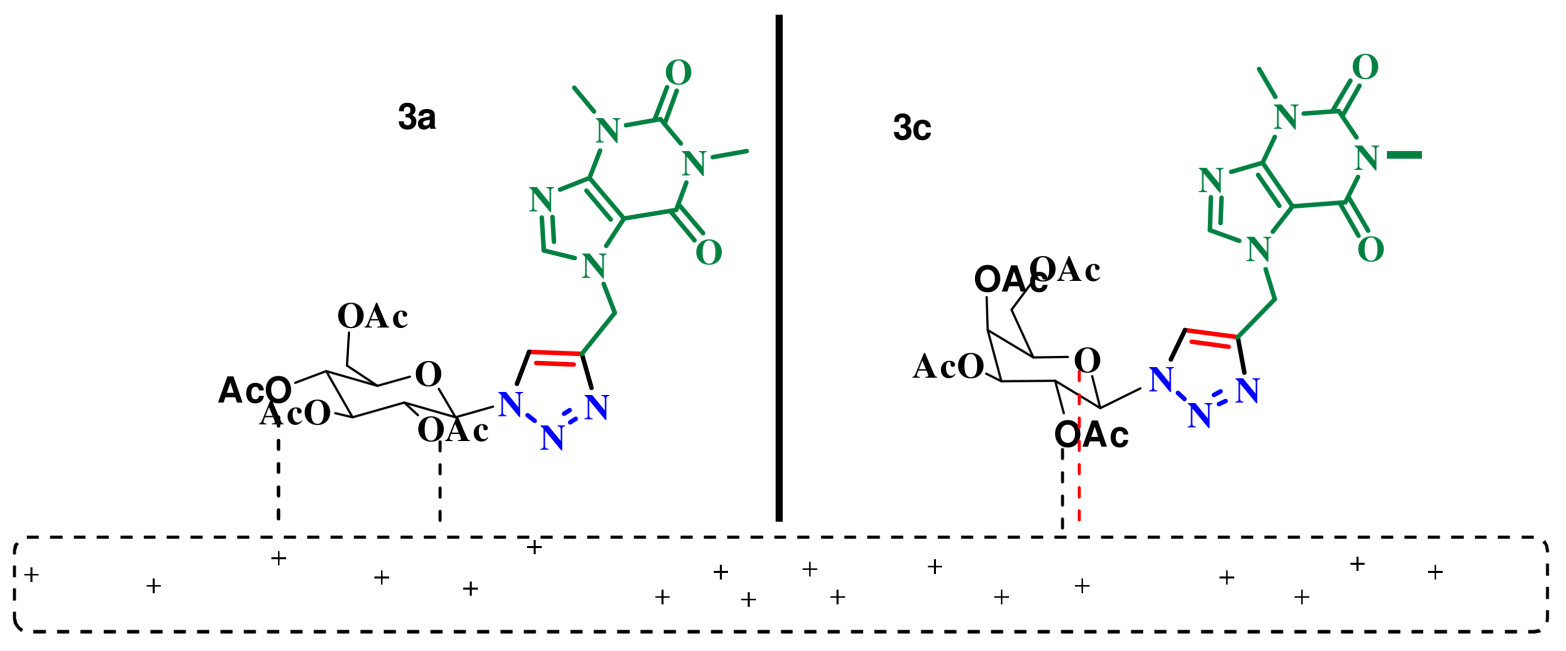
Comparison of inhibitor adsorption by compound **3a** and **3c** in metal surface.

In this context, we suggest that a much stronger contribution provided by oxygen “as an active center with excess charge” within the pyranoic ring is favored in **3c** and consequently has a better interaction with the metal surface, while in **3a** the electronic contribution is not favored, being only a minor contribution by the Oacs (Figure 9).

#### 2.2.6. Surface Morphology by SEM-EDS

The SEM micrographics were performed to complete the results obtained by EIS in the absence and presence of the best concentration of **3a** and **3c** (20 ppm) immersed in HCl 1 M.

**Figure 10:**
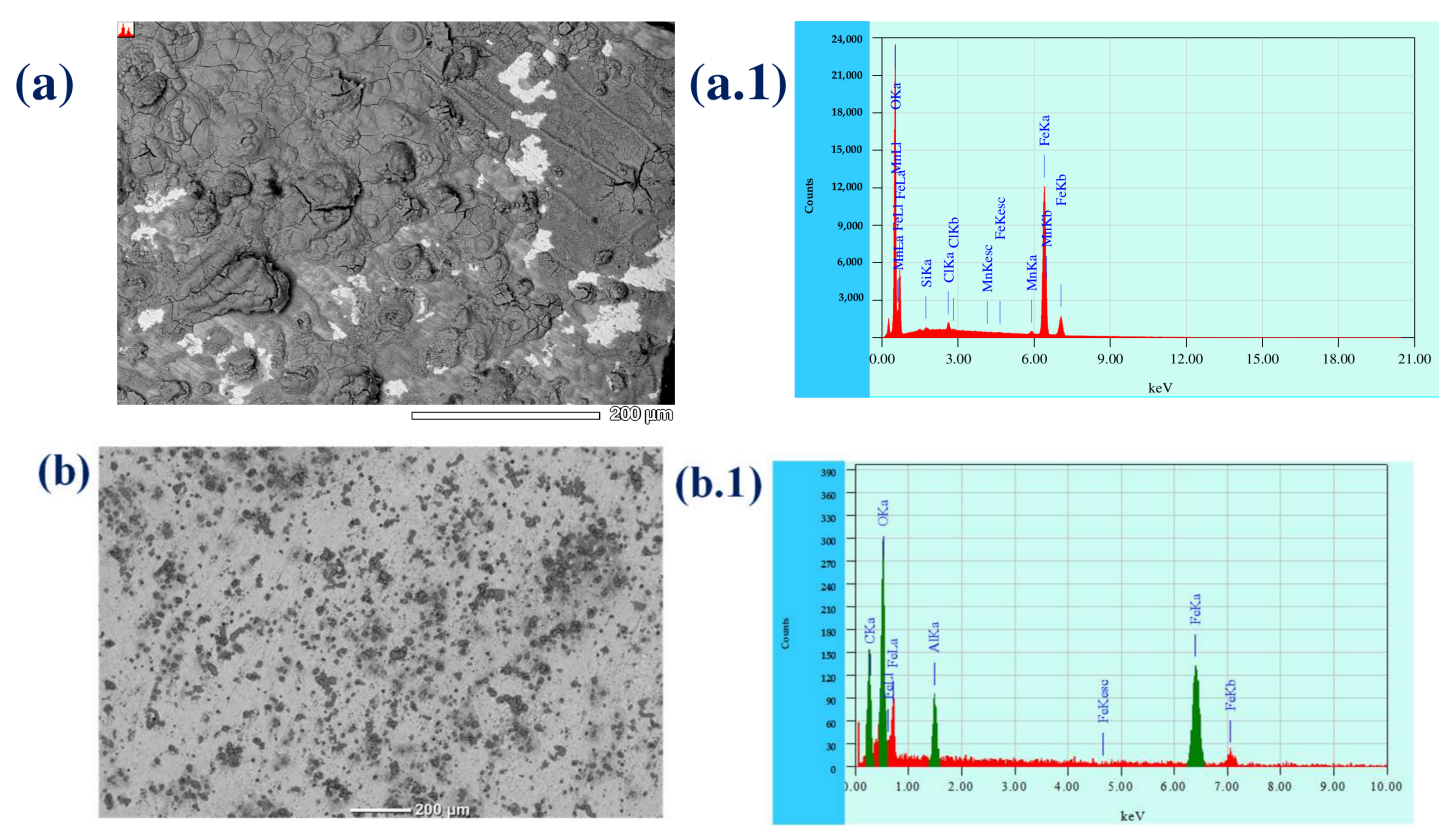

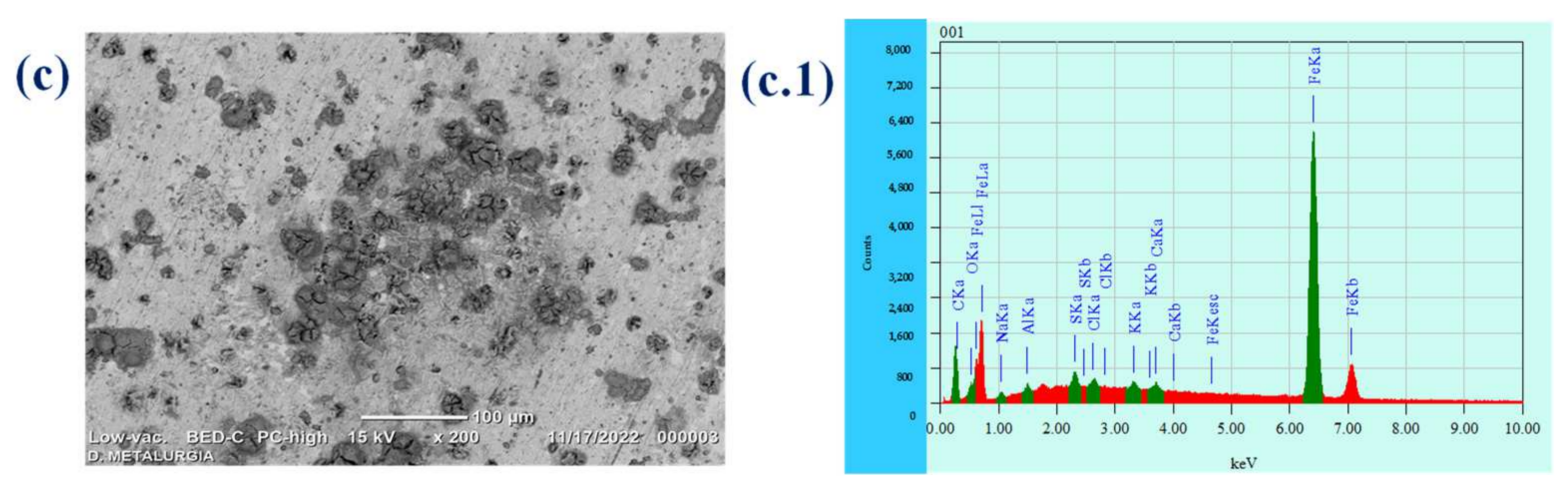
SEM-EDS images at 200× of (**a**,**a.1**) API 5L X70 steel with no inhibitor, (**b**,**b.1**) API 5L X70 steel + 20 ppm with **3b** and (**c**,**c.1**) API 5L X70 steel + 20 ppm with **3f**, immersed in HCl 1 M after 24 h.

In Figure 10a, it is shown that the surface was damaged in the absence of the organic compound, and it also presents several corrosion products. Then, in Figure 10b,c, the surface appears less damaged in the presence of **3a** and **3c** inhibitors compared to the surface immersed in the corrosive medium by itself.

By using the chemical analysis performed for each sample (Figure 10a.1–c.1), it was possible to observe that, in the case of the metal immersed in the corrosive medium, there is the presence of the corrosive species chlorine and oxygen (Figure 10a.1).

The damage on the metallic surface has decreased in the presence of the organic compound, as the presence of chloride and oxygen ions has decreased, which is attributed to the formation of a protective film on the active sites where the organic molecules are present with pairs of free electrons (Table 4). Finally, carbon was detected as a result of the presence of organic molecules.

#### 2.2.7. AFM Analysis

**Figure 11:**
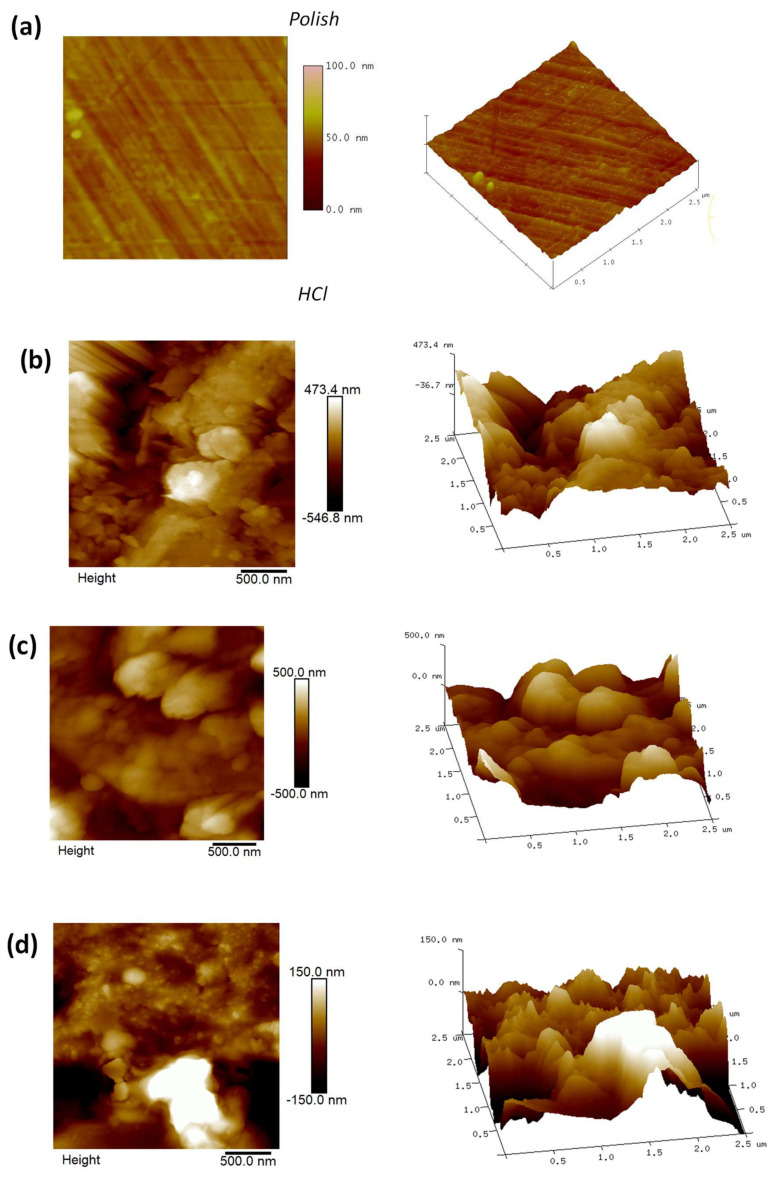
AFM images at different conditions (**a**) polish, (**b**) steel immersed in HCl 1 M, (**c**) inhibitor **3b** and (**d**) inhibitor **3f**.

The AFM images in Figure 11 and surface roughness are indicators of the corrosion grade on the metal surface. When the metal is polished (Figure 11a), the roughness of the metal is very low compared to the metal exposed to the corrosive medium (Figure 11b). In the presence of inhibitors **3b** and **3f**, the AFM images shown in Figure 11c,d are covered by a thin film and appear rather denser and smoother. This presumably resulted in the formation of an inhibitor adsorption film on the surface of the steel sample [45]. Moreover, the parameters of average roughness (Ra) and root-mean-square roughness (Rq) values are summarized in Table 5. It is very clear that the values of Ra and Rq decreased with the presence of the inhibitor corrosion film.

#### 2.2.8. Theoretical Assessment

The presence of nitrogen as a heteroatom in the inhibitor molecules provides a high tendency to protonation in an aqueous acidic medium (HCl 1 M). Under these conditions, the calculations accounted for the complete set of electrons, and the geometry of the involved structures was fully optimized. This means that the nitrogen atoms hold a positive charge with the addition of hydrogen atoms, and the solvent (water) effect is included within the solvation model based on density (SMD) [46].

The electronic structure of the six molecules considered in this work was determined through the Gaussian09 suite [47], and all calculations were carried out using the B3LYP functional [48,49] with the 6–311++G orbital basis set. Full geometry optimizations were performed for all the cases considered, followed up by frequency calculations to ensure stability. The optimized geometries of the compounds before protonation are presented in Figure 1. A crucial fact in the adsorption over the metallic surface is the planar configuration of the inhibitor molecules. The frameworks of these geometries show no planar configurations for any inhibitors. Since, under experimental conditions, the prototype inhibitors are protonated, we have carried out a complete protonated structure in all possible sites (heteroatom positions, nitrogen) because, at the experimental pH of 0, multiple protonation should be expected to occur. The results for the optimized geometries of the protonated species are presented in Figure 1, where it is possible to see that they are not planar.

**Figure 12:**
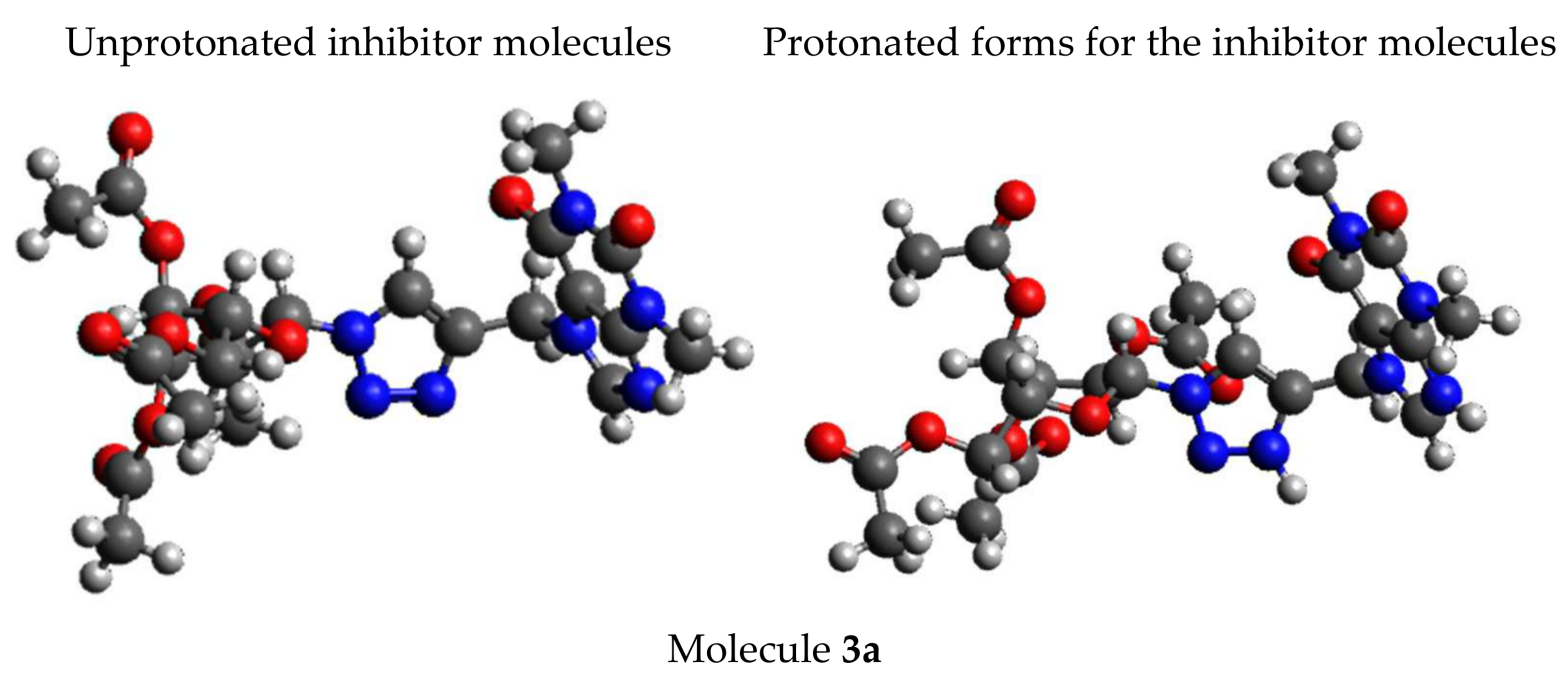

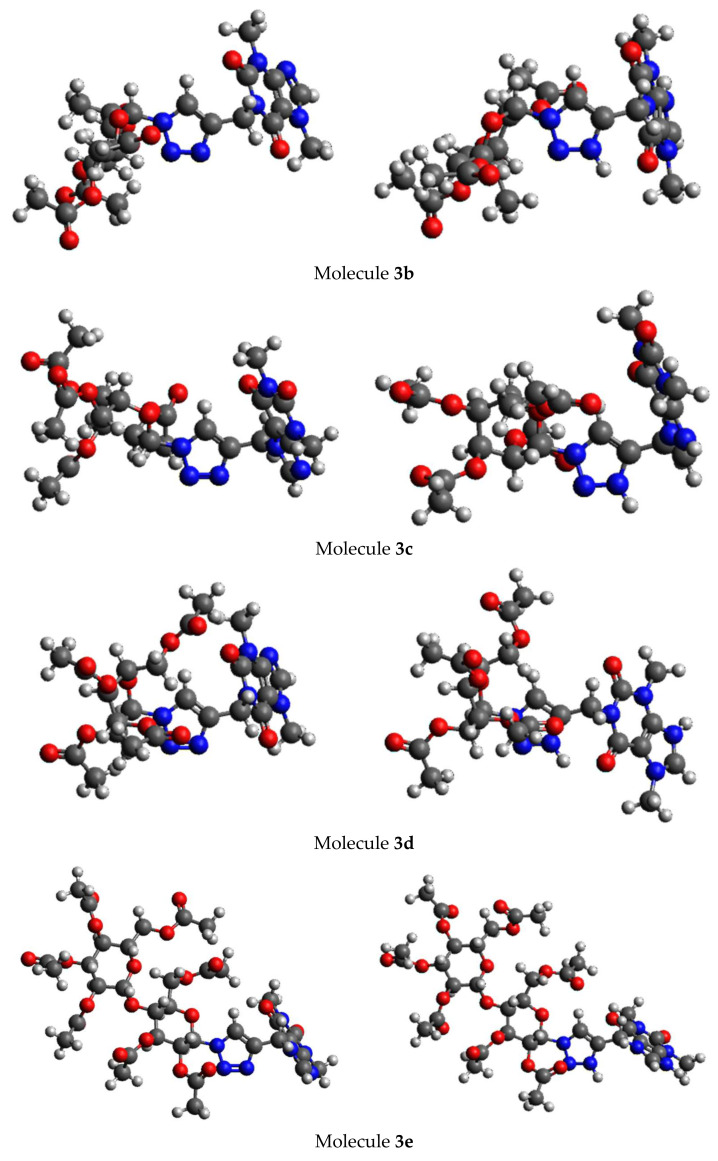

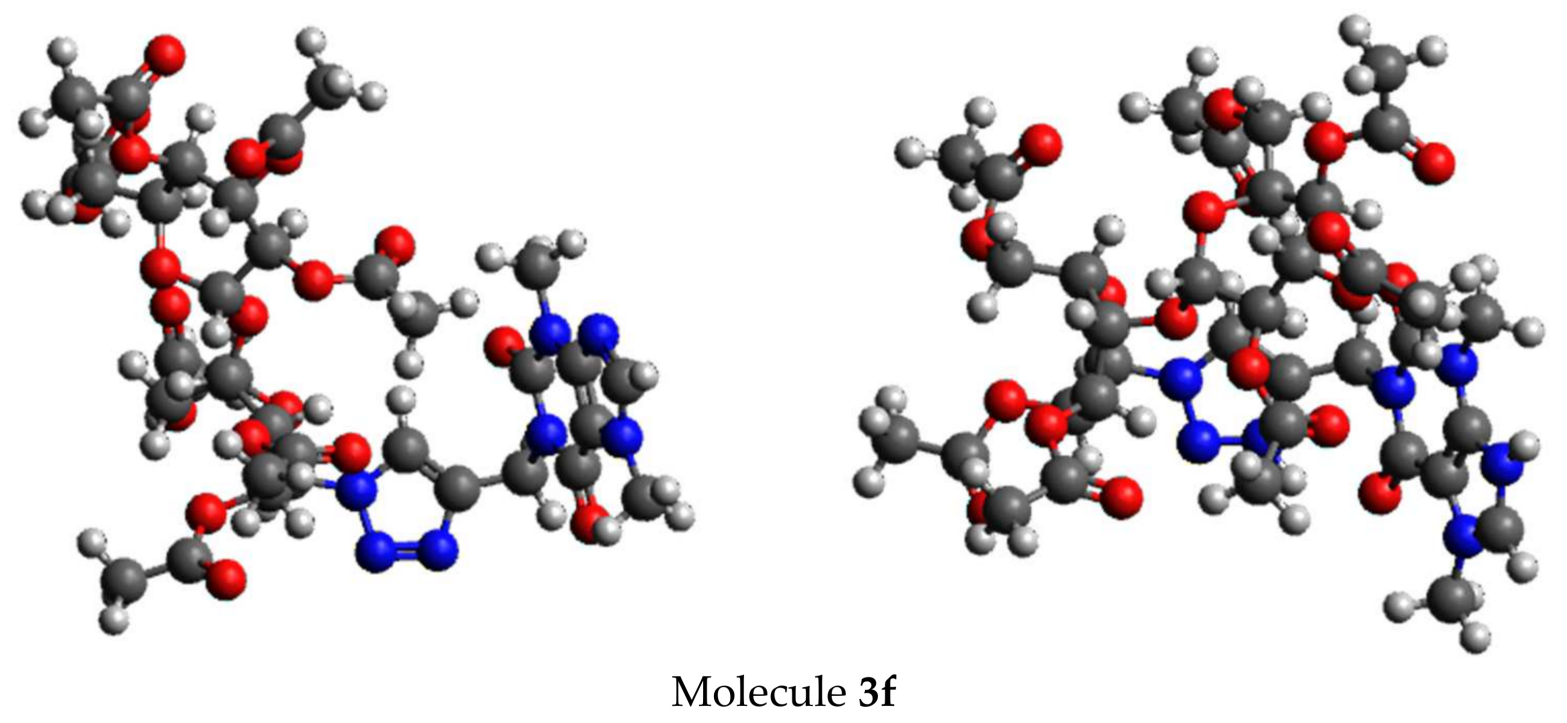
Optimized unprotonated and protonated inhibitor molecules considered in the present study. Gray corresponds to carbon, blue to nitrogen, red to oxygen, and white to hydrogen.

**Figure 13:**
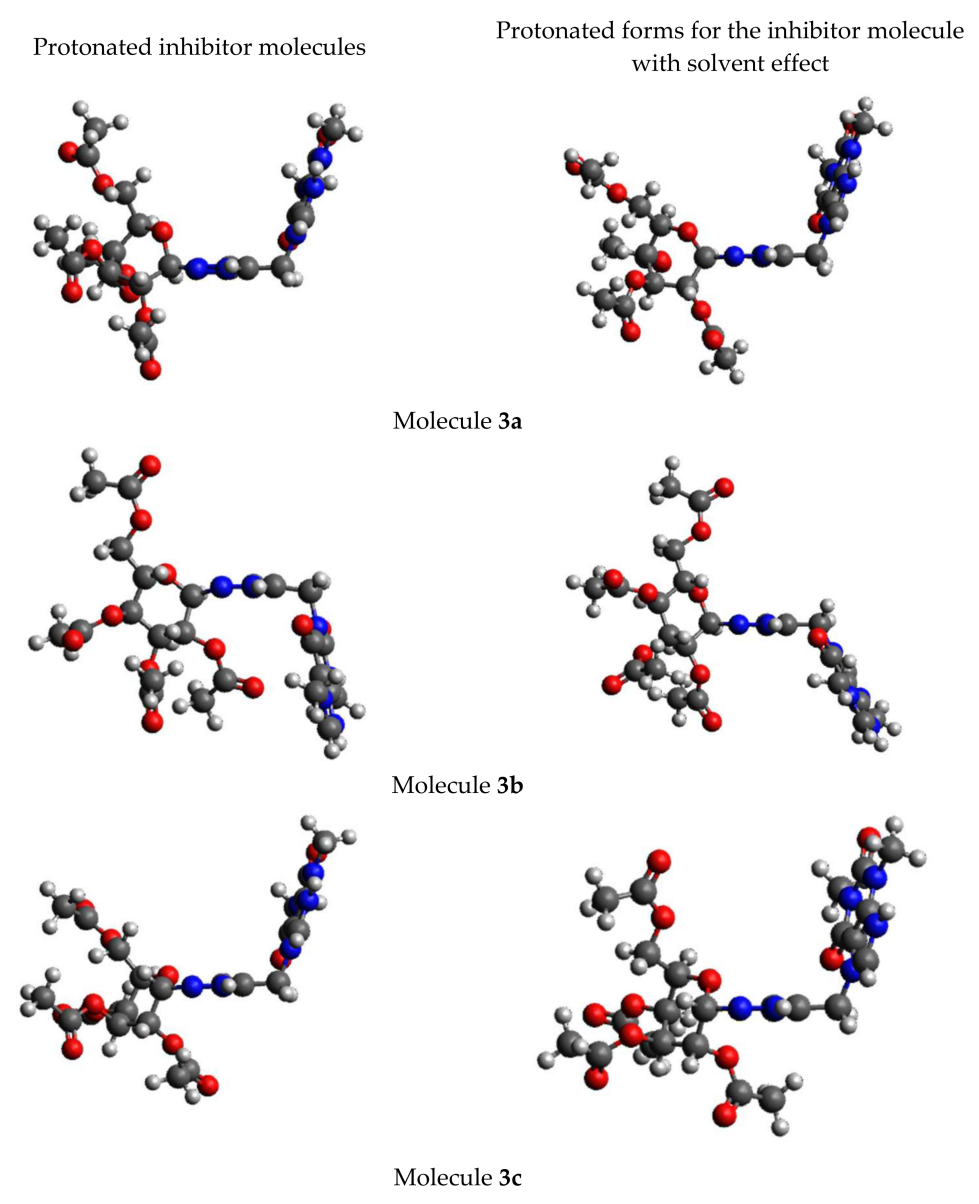

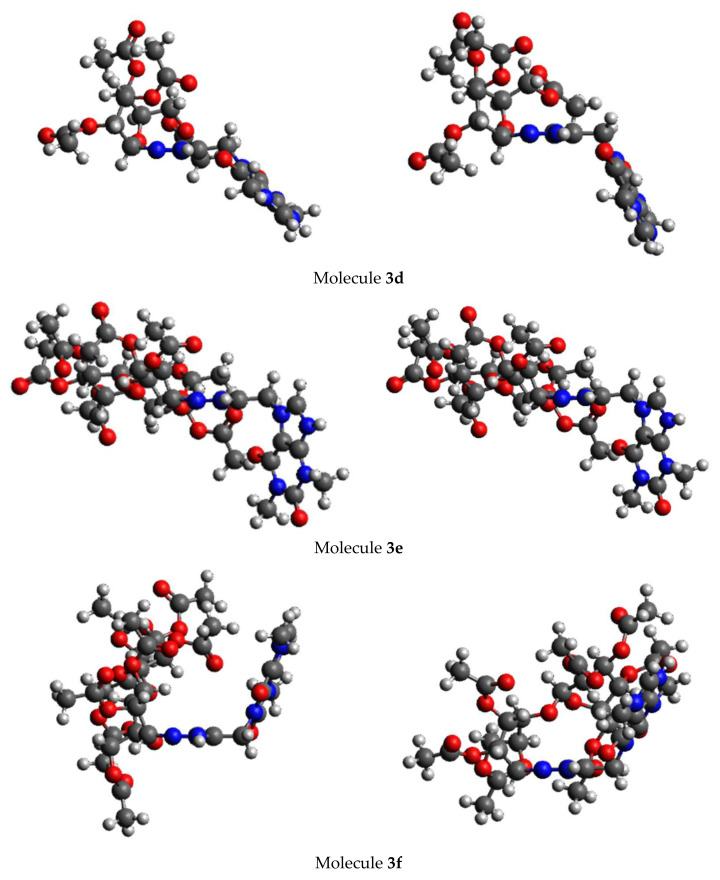
Optimized protonated inhibitor molecules without and with solvent effect.

From Figure 12, we can observe that the presence of protons changes the geometrical structure; in some cases, these changes are very strong. For example, in molecule **3f**, lactose moiety moves towards theobromine, folding the structure. It is important to note that, under testing conditions, the inhibitors are in their protonated forms and have a solvent effect. In Figure 13, we present how the protonated structures change when the solvent is included. We observe that the solvent induces an increase in the folding of geometry.

It can be observed in Figure 12 and Figure 13 that both the unprotonated molecules and the protonated ones do not show a high degree of planarity; only the theophylline and theobromine moieties kept planarity, which led to a high electronic delocalization, typical of a resonant system. This situation indicates that the molecular interaction with the metallic surface possibly occurs through the rings of theophylline and theobromine.

For this set of molecules, a way to predict the most favorable site to predict their reactivity is to employ molecular orbital theory.

**Figure 14:**
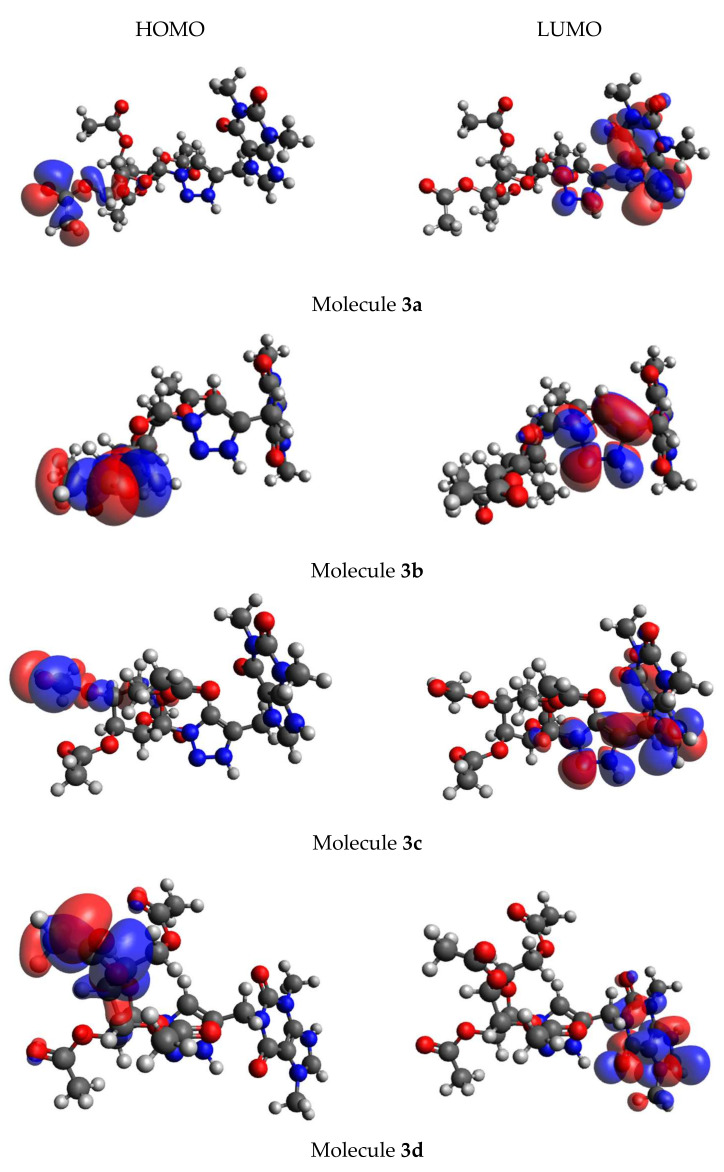

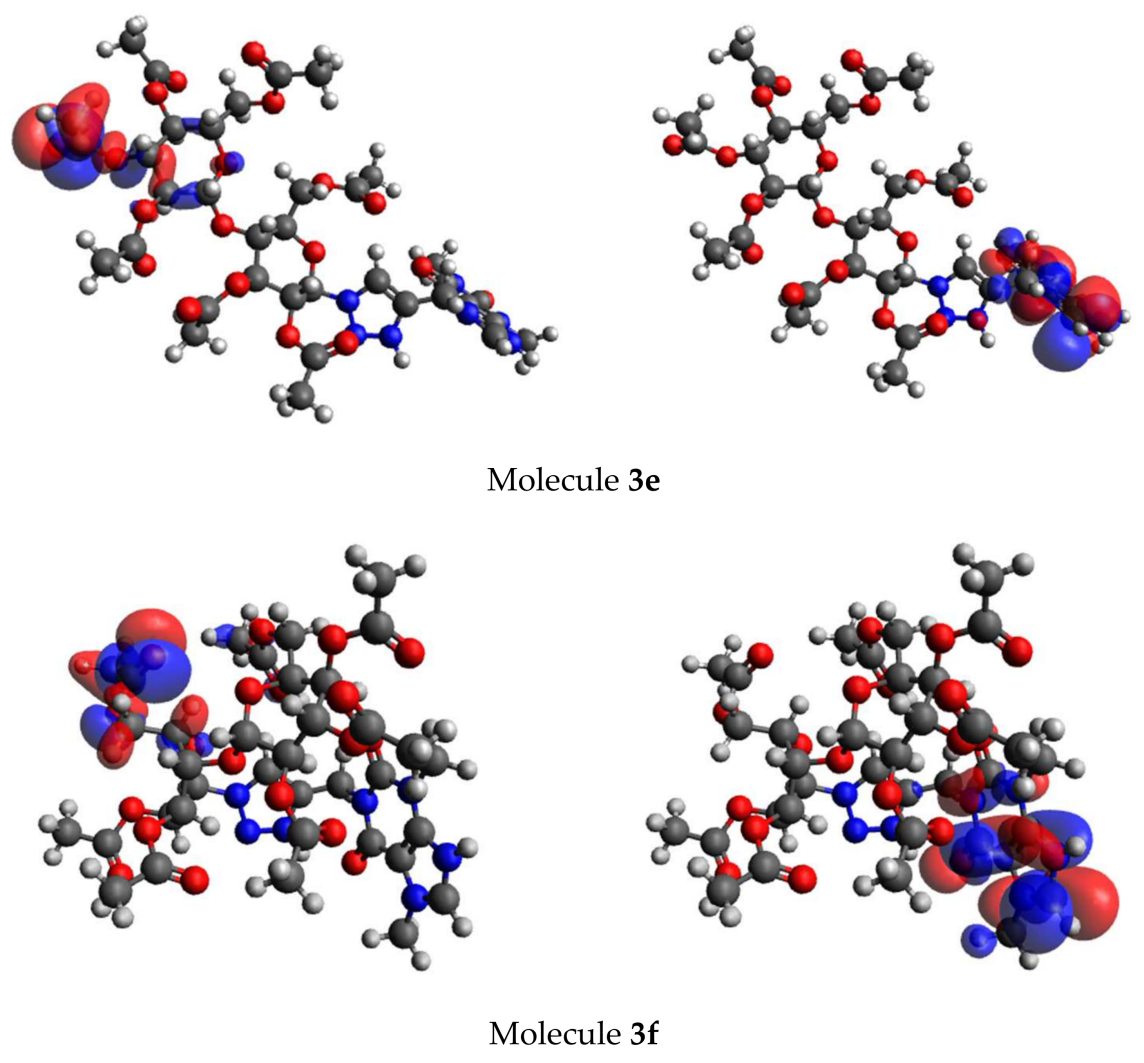
Representation of the frontier orbitals, HOMO and LUMO, of the set of molecules.

In Figure 14, we can see the representation of the frontier molecular orbitals. The donor character, HOMO, is localized at carbohydrate for the entire set, while the acceptor character, LUMO, is mainly localized at xanthine for **3d**, **3e** and **3f**. For **3a** and **3c**, LUMO is also in the triazole, and in **3b**, LUMO is just in the triazole.

Moreover, global reactivity indexes were calculated to assess how they performed compared to other species. Particularly, the nucleophilicity index (N) was analyzed. Domingo et al. [50] proposed an empirical (relative) nucleophilicity N index for organic molecules based on the HOMO energies, EHOMO, obtained within the Kohn–Sham scheme and defined as follows:

N = EHOMO (Nucleophile) − EHOMO(TCE)

This N index referred to tetracyanoethylene (TCE), which is the most electrophilic neutral species in a tested set. Strong nucleophiles are considered when N is higher than 3.0 eV, moderate with 2.0 ≤ N ≤ 3.0 eV, and marginal with N < 2.0 eV. Table 6 shows N index values, and we can conclude that this family performs as moderate nucleophiles; molecule **3f** is considered a strong nucleophile; these characteristics point out that this family would be a good corrosion inhibitor. It is worthy to observe the lowest N values for molecules **3a** and **3d**: experimental results show that molecule **3a** presents the worst performance in the set, while molecule **3d** is the best.

A good inhibitor can be identified for its spatial, molecular, and electronic structure. Some quantum-chemical parameters can be correlated with metal-inhibitor interactions. The HOMO energy, EHOMO, suggests the electron donation ability of the inhibitor molecule towards the metallic surface atoms, and it is expected that a higher EHOMO value would favor a greater charge transfer, thus the molecule will be a good inhibitor of corrosion [51,52].

According to our results (see Table 7), the obtained values of EHOMO for each molecule present a small difference among them, highlighting a very similar capacity of electron donation on the metallic surface. In the protonated tested set, Molecule **3e** shows the best electron donation ability, and the worst would be Molecule **3c**; however, in a solvent (liquid phase), we can observe that the differences among them are little. Bentiss et al. [53] establish that molecules with low gap energy (ΔE) provide good inhibition efficiencies, as the necessary excitation energy to remove an electron from HOMO will be less. Table 7 shows the gap energy values for the tested molecules.

From Table 8, we observe a small ΔE value for molecule **3e**, which indicates the best performance as an inhibitor. In a liquid medium, the differences are small among them and support the good efficiency that they display. Gómez et al. [54], proposed that the interaction between the inhibitor and the metallic surface occurs through donation and back-donation; they used a simple charge transfer model and established that the interaction energy is favored when hardness increases. If we assume that the interaction of these molecules with a metal surface occurs through donation and back-donation and that the inhibitor efficiency should increase when there is better adsorption of the molecule to the metal surface, then the inhibition efficiency should increase when the stabilization energy that results from the interaction between the metal surface and the inhibitor increases. Table 9 presents the chemical hardness values (η) for protonated and unprotonated tested molecules.

**Figure 15:**
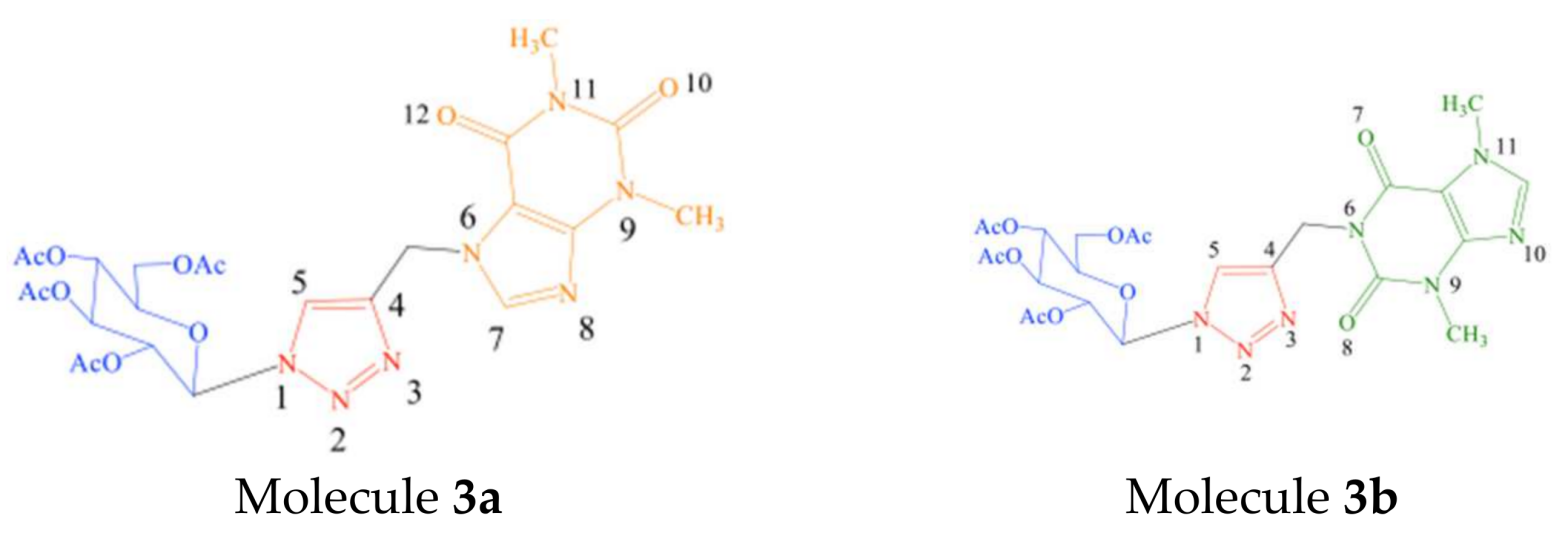
Referenced atoms in Table 5.

The results in Table 9 show that unprotonated molecules are better inhibitors than protonated forms. Unprotonated molecules follow the order **3d** > **3b** > **3e**, **3f** > **3c** > **3a**, which agrees with the efficiency experimentally found, while for protonated molecules it is **3f** > **3c** > **3d** > **3b** > **3a** > **3e**. When the solvent effect is considered over the protonated molecules, it recovers the experimental order **3d**, **3f** > **3b**, **3e** > **3c** > **3a**. We should mention that hardness values are very close among them. The Hirshfeld atomic charges for the most important centers in protonated molecules are summarized in Table 10 (also see Figure 15). We can observe that all the atoms in the triazol ring present a positive charge, while oxygen and two nitrogen atoms on the theophylline and theobromine groups have a negative charge; also, all the oxygen atoms present in carbohydrate, glucose, galactose, and lactose show a negative charge. This fact suggests that those are active centers with excess charges that could act as a nucleophile group. The most favorable sites for the interaction with the metal surface are the oxygen atoms, which have the largest negative charges of all the tested molecules.

**Figure 16:**
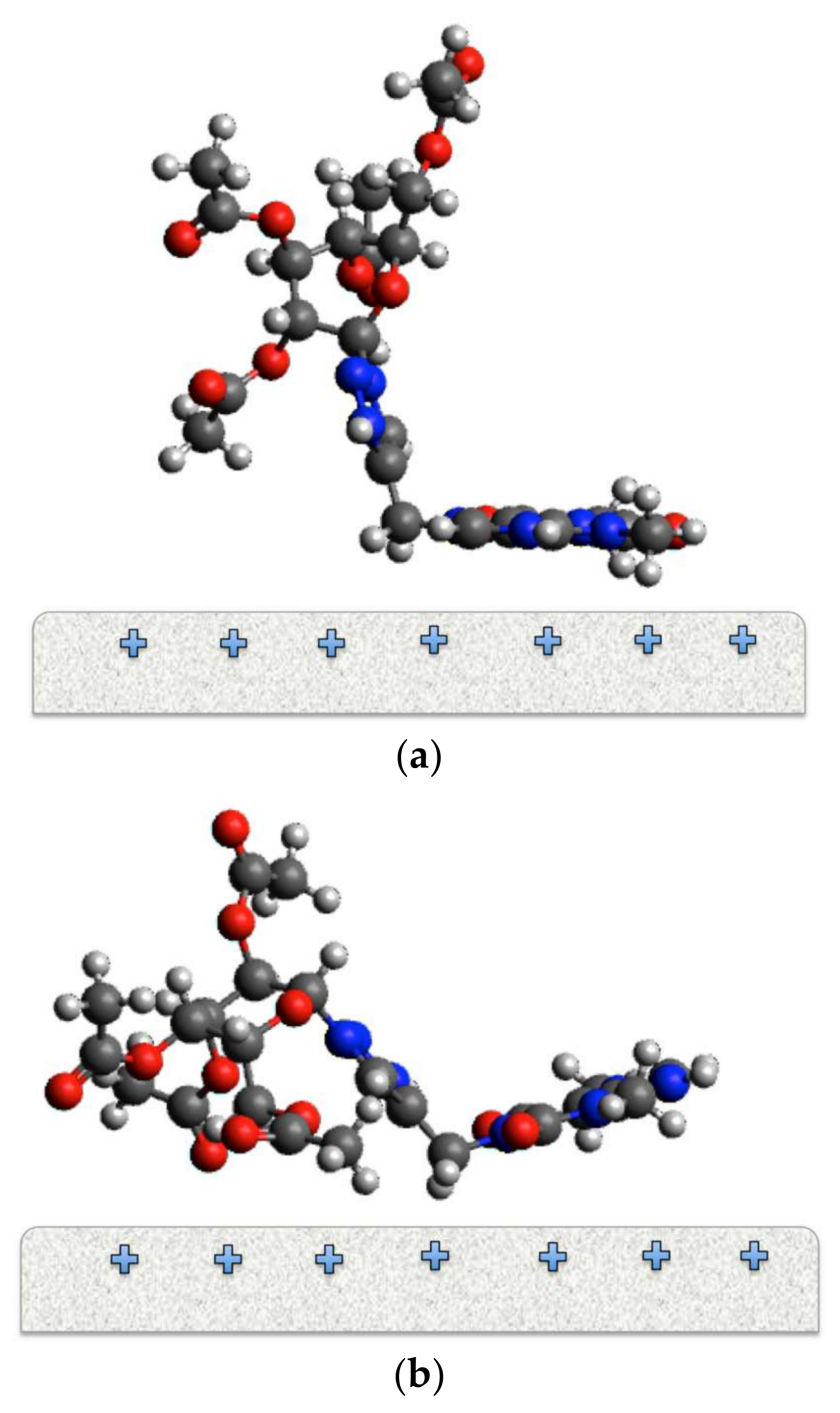
Presumable molecular adsorptions for, (**a**) Molecule **3a** and (**b**) Molecule **3d**.

From the evidence given above, we can suggest that, due to the planar geometry of theophylline, the molecular adsorption of molecule **3a** probably occurs in such a way, as shown in Figure 16a, and if the interaction between the molecule and the metallic surface occurs through donation and back-donation, as Hirshfeld charges support the donation and back-donation mechanism, this molecule is the softest and therefore the worst inhibitor in the set [55]. While molecule **3d**, the best inhibitor in the tested set, is the hardest molecule and presents a certain planarity, theobromine and galactose moieties with negative charges in nitrogen and oxygen atoms can increase the interaction inhibitor–metallic surface through a parallel arrangement (see Figure 16b).

Quantum chemical calculations revealed that it is necessary to include experimental conditions such as acidic media, which means protonated molecules in this case, and the effect of solvent. Under these conditions, the 3D structures of the six molecules studied were visualized, and they were not planar, which makes the adsorption over a surface more difficult. The nucleophilicity index N shows that the set of molecules perform as moderate nucleophiles; molecule **3f** is the best nucleophile in correlation with experimental results, while molecules **3a** and **3d** are the worst nucleophiles; however, molecule **3d** is one of the best inhibitors in the set according to experimental evidence. The HOMO energy is very similar among the six molecules; this result does not indicate that molecule **1**, presents a low-efficiency inhibitor. In the same way, the HOMO-LUMO gap energy, ΔE, is very similar among all the sets of molecules. Efficiency of inhibitor molecules cannot be directly correlated to individual molecular parameters. Chemical hardness values η, recover the experimental inhibition efficiency order for protonated molecules with a solvent effect. Then the interaction molecule—metallic surface occurs through donation and back-donation. This fact is supported by Hirshfeld atomic charges that reveal that the adsorption of molecules studied is mainly concentrated around the oxygen atoms (heteroatom), specifically in theophylline and theobromine moieties. Molecules that show less folding could interact with metallic surfaces through donation and back-donation by theobromine/theophylline and carbohydrate moieties at sites where the oxygen atoms have excess charge.

## 3. Materials and Methods

### 3.1. Synthesis

All reagents purchased commercially were used without purification. The solvents were of technical grade and freshly distilled before use unless otherwise noted. Melting points were obtained on a Fisher-Johns apparatus and are uncorrected. NMR spectra were recorded on Bruker (Billerica, MA, USA) Ascend-400 (400 MHz) and Bruker Avance DMX-400 (400 MHz) spectrometers in CDCl_3_, and chemical shifts are given in ppm, multiplicity (s = singlet, d = doublet, t = triplet, q = quartet, m = multiplet, br s = broad singlet), coupling constant (J) with TMS as the reference. The MS-DART data were obtained on a Jeol AccuTOF (Tokyo, Japan); the values of the signals are expressed as mass/charge units (*m*/*z*). Microwave irradiation experiments were performed using a Discover System (CEM Corporation, Matthews, NC, USA) single-mode microwave with standard sealed microwave glass vials.

#### 3.1.1. Synthesis of Carbohydrate-Xanthine Conjugates

A mixture of catalyst (10 mg) and EtOH-H_2_O (2 mL, 3:1 *v*/***v***) was placed in a microwave tube with a magnetic stirrer. Subsequently, propargyl xanthines **2a**–**b** (1 mmol), azido-sugars **1a**, **1b,** or **1c** (1.2 mmol), and sodium ascorbate (0.01 mmol) were added to the mixture, which was heated under microwave irradiation (40 W, 80 °C) for 30 min. Then, the reaction mixture was diluted with dichloromethane and filtered through a short plug of silica. The organic extract was dried with Na_2_SO_4_, and the solvent was evaporated; the resultant residue was purified by column chromatography.

#### 3.1.2. Synthesis of Glucose-Triazole-Theophylline Conjugate (**3a**)

The title compound was prepared from glucosylazide **1a** (0.100 g) according to general procedure B to afford, after purification by column chromatography (ethyl acetate), compound **3a** in a 73% yield (0.115 g) as a white solid: m.p. = 98–99 °C, [α]_D_^25^ = −17.0 (c 1, CHCl_3_). ^1^H NMR (400 MHz, CDCl_3_) δ 8.05 (s, 1H), 5.84 (m, 1H), 5.63(d, *J* = 14.8 Hz, 1H), 5.58 (d, *J* = 14.8 Hz, 1H), 5.46–5.34 (m, 2H), 5.29–5.20 (m, 1H), 4.30 (dd, *J* = 12.7, 4.8 Hz, 1H), 4.20–4.11 (m, 1H), 3.99 (ddd, *J* = 10.2, 4.9, 2.1 Hz, 1H), 2.08 (s, 3H), 2.07 (s, 3H), 2.03 (s, 3H), 1.80 (s, 3H). ^13^C NMR (101 MHz, CDCl_3_) δ 170.62, 170.05, 169.46, 168.84, 155.56, 151.78, 149.05, 142.95, 141.29, 122.50, 106.68, 85.95, 77.48, 77.16, 76.84, 75.35, 72.53, 70.42, 67.64, 61.50, 60.55, 53.58, 41.52, 31.72, 29.94, 28.18, 20.84, 20.67, 20.65, 20.21. HR-MS (ESI-TOF) calculated for C_36_H_46_N_7_O_19_ [M + H]^+^ 592.20033, found 592.19945.

#### 3.1.3. Synthesis of Glucose-Triazole-Theobromine Conjugate (**3b**)

The title compound was prepared from glucosylazide **1a** (0.100 g,) according to general procedure B to afford, after purification by column chromatography (ethyl acetate), compound **3b** in 70% yield (0.110 g) as a white solid: m.p = 112–115 °C, [α]_D_ ^25^ = −24.4 (c 1, CHCl_3_). ^1^H NMR (500 MHz, CDCl_3_) δ 7.85 (s, 1H), 7.51 (d, *J* = 0.6 Hz, 1H), 5.85 (d, *J* = 9.0 Hz, 1H), 5.46–5.36 (m, 3H), 5.27 (d, *J* = 12 Hz, 1H), 5.22 (dd, *J* = 10.1, 9.2 Hz, 1H), 4.28 (dd, *J* = 12.6, 5.0 Hz, 1H), 4.16–4.11 (m, 1H), 4.00 (d, *J* = 0.6 Hz, 3H), 3.99–3.96 (m, 1H), 3.57 (s, 3H), 2.08 (s, 3H), 2.06 (s, 3H), 2.02 (s, 3H), 1.84 (s, 3H). ^13^C NMR (126 MHz, CDCl_3_) δ 170.62, 170.02, 169.42, 168.94, 154.88, 151.39, 149.14, 144.48, 141.74, 121.92, 107.76, 85.76, 75.23, 72.92, 70.38, 67.85, 61.68, 36.01, 33.74, 29.88, 20.81, 20.64, 20.62, 20.30. HR-MS (ESI-TOF) calculated for C_36_H_46_N_7_O_19_ [M + H]^+^ 592.20033, found 592.19748.

#### 3.1.4. Synthesis of Galactose-Triazole-Theophylline Conjugate (**3c**)

The title compound was prepared from galactosylazide **1b** (0.100 g) according to general procedure B to afford, after purification by column chromatography (ethyl acetate), compound **3c** in 69.5 % yield (0.116 g) as a white solid: m.p.= 88–90 °C, [α]_D_ ^25^ = −8.0 (c 1, CHCl_3_). ^1^H NMR (400 MHz, CDCl_3_) δ 8.07 (s, 1H), 5.80 (d, *J* = 9.2 Hz, 1H), 5.63 (q, *J* = 15.1 Hz, 2H), 5.57–5.53 (m, 1H), 5.48 (dd, *J* = 10.3, 9.2 Hz, 1H), 5.23 (dd, *J* = 10.3, 3.4 Hz, 1H), 4.26–4.01 (m, 3H), 3.58 (s, 3H), 3.44 (s, 3H), 2.24 (s, 3H), 2.05 (s, 3H), 2.01 (s, 3H), 1.84 (s, 3H). ^13^C NMR (101 MHz, CDCl_3_) δ 170.32, 169.98, 169.79, 168.94, 155.45, 151.62, 148.86, 142.62, 141.21, 122.26, 106.54, 86.39, 74.16, 70.52, 67.90, 66.68, 61.12, 41.31, 29.80, 28.01, 20.69, 20.63, 20.48, 20.17. HR-MS (ESI-TOF) calculated for C_36_H_46_N_7_O_19_ [M + H]^+^ 592.20033, found 592.19957.

#### 3.1.5. Synthesis of Galactose-Triazole-Theobromine Conjugate (**3d**)

The title compound was prepared from galactosylazide **1b** (0.100 g) according to general procedure B to afford, after purification by column chromatography (ethyl acetate), compound **3c** in 74% yield (0.116 g) as a white solid: m.p. = 115–117 °C, [α]_D_^25^ = −28.0 (c 1, CHCl_3_). ^1^H NMR (500 MHz, CDCl_3_) δ 7.92 (s, 1H), 7.52 (d, *J* = 0.6 Hz, 1H), 5.83 (d, *J* = 9.4 Hz, 1H), 5.58–5.52 (m, 2H), 5.41 (d, *J* = 14.6 Hz, 1H), 5.25 (d, *J* = 14.3 Hz, 1H), 5.23–5.18 (m, 1H), 4.25–4.09 (m, 4H), 4.00 (s, 3H), 3.58 (s, 3H), 2.24 (s, 3H), 2.04 (s, 3H), 2.00 (s, 3H), 1.85 (s, 3H). ^13^C NMR (101 MHz, CDCl_3_) δ 170.5, 170.2, 170.0, 169.17, 154.9, 151.4, 149.11, 144.31, 141.76, 122.07, 107.76, 86.3, 74.13, 71.01, 67.85, 66.96, 61.35, 35.95, 31.8, 29.9, 20.89, 20.81, 20.65, 20.44. HR-MS (ESI-TOF) calculated for C_36_H_46_N_7_O_19_ [M + H]^+^ 592.20033, found 592.19759.

#### 3.1.6. Synthesis of Lactose-Triazole-Theophylline Conjugate (**3e**)

The title compound was prepared from lactozylazide **1c** (0.100 g) according to general procedure B to afford, after purification by column chromatography (ethyl acetate), compound **3e** in a 65% yield (0.086 g) as a white solid: m.p. = 140–143 °C. [α]_D_ ^25^ = −16.00 (c 1, CHCl_3_). ^1^H NMR (400 MHz, Chloroform-d) δ 7.98 (s, 1H), 7.78 (s, 1H), 5.78 (d, *J* = 8.9 Hz, 1H), 5.64–5.49 (m, 2H), 5.45–5.26 (m, 3H), 5.11 (dd, *J* = 10.4, 7.8 Hz, 1H), 4.96 (dd, *J* = 10.4, 3.4 Hz, 1H), 4.49 (dd, *J* = 13.3, 10.5 Hz, 2H), 4.19–4.02 (m, 3H), 4.01–3.85 (m, 3H), 3.55 (s, 3H), 3.40 (s, 3H), 2.14 (s, 3H), 2.09 (s, 3H), 2.06 (s, 3H), 2.04 (d, *J* = 3.2 Hz, 6H), 1.95 (s, 3H), 1.77 (s, 3H).^13^C NMR (101 MHz, CDCl_3_) δ 170.45, 170.24, 170.19, 170.14, 169.54, 169.14, 169.04, 155.54, 151.71, 149.00, 141.27, 122.59, 106.64, 101.21, 85.76, 76.13, 75.58, 72.45, 70.99 (2C), 70.67, 69.14, 66.69, 61.66, 60.94, 41.52, 29.95, 29.78, 28.12, 20.90, 20.78 (2C), 20.73 (2C), 20.60, 20.23. HR-MS (ESI-TOF) calculated for C_36_H_46_N_7_O_19_ [M + H]^+^ 880.28315, found 880.28485.

#### 3.1.7. Synthesis of Lactose-Triazole-Theobromine Conjugate (**3f**)

The title compound was prepared from lactozylazide **1c** (0.100 g) according to general procedure B to afford, after purification by column chromatography (ethyl acetate), compound **3f** in a 71% yield (0.091 g) as a white solid: m.p. = 149–131 °C, [α]_D_ ^25^ = −19.0 (c 1, CHCl_3_). NMR (500 MHz, CDCl_3_) δ 7.82 (s, 1H), 7.51 (s, 1H) 5.81 (d, *J* = 9.0 Hz, 1H), 5.44–5.35 (m, 3H), 5.27 (d, *J* = 14.6 Hz, 1H), 5.13 (dd, *J* = 10.4, 7.9 Hz, 1H), 4.99 (dd, *J* = 10.4, 3.5 Hz, 1H), 4.54 (d, *J* = 7.9 Hz, 1H), 4.47 (dd, *J* = 12.2, 1.8 Hz, 1H), 4.17–4.08 (m, 2H), 4.0 (s, 2H), 3.98–3.86 (m, 2H), 3.57 (s, 3H), 2.16 (s, 3H), 2.11 (s, 3H), 2.08 (s, 3H), 2.06 (s, 3H) 2.05 (s, 3H), 1.97 (s, 3H), 1.83 (s, 3H). ^13^C NMR (126 MHz, CDCl_3_) δ 170.50, 170.40, 170.20, 170.10, 169.60, 169.04, 169.03, 154.85, 151.40, 149.00, 144.22, 141.70, 130.84, 122.14, 107.70, 101.20, 85.50, 75.90, 75.70, 72.80, 71.00, 70.90, 70.60, 69.15, 66.70, 61.80, 60.98, 35.92, 33.74, 29.80, 20.93, 20.78 (2C), 20.73 (2C), 20.61, 20.40. HR-MS (ESI-TOF) calculated for C_36_H_46_N_7_O_19_ [M + H]^+^ 880.28485 found 880.28568.

### 3.2. Electrochemical Evaluation

According to previous works, the steel API 5L X70 was prepared by sanding with sandpaper from 220, 320, 400, and 600 grid. Afterwards, the dissolution of 0.01 M of inhibitors **3a**–**3f** dissolved in ethanol was prepared. Subsequently, concentration sweeps of 5, 10, 20, and 50 ppm of the inhibitor in the corrosive solution of HCl 1 M were performed. The electrochemical cell used in this paper is described as follows: the working electrode was API 5L X70 steel with an exposed area of 0.76 cm^2^, the reference electrode was Ag/AgCl saturated, and the counter electrode was graphite.

The potential was stabilized until 1800 s, when it reached a plateau. By electrochemical impedance spectroscopy technique, a sinusoidal potential of ±10 mV in a frequency interval (100 KHz to 0.1 Hz) was applied in a three-electrode arrangement. The polarization curves of the inhibitor at different concentrations were performed, which were measured from −500 mV to 500 mV in relation to the open circuit potential (OCP) at a speed of 60 mV/min using the ACM Analysis software for data interpretation.

### 3.3. Characterization by SEM-EDS and AFM

The surface of the samples was analyzed by scanning electron microscopy (SEM), using a JCM5000PLUS at 10 kV. The chemical analysis of the resulting corrosive products was performed by energy-dispersive X-ray spectroscopy (EDS) in a timely manner with a time spectrum acquisition of 30%. Finally, the studies of morphology were imaged via a Digital Instruments Scanning Probe Microscope, using an atomic force microscope (AFM) with a NanoScope IIIa controller. The AFM was operated in tapping mode using an etched silicon cantilever with a length of 125 μm and a nominal tip radius of approximately 10 nm.

## 4. Conclusions

In this work, the corrosion inhibition properties of six organic molecules synthesized and obtained in good reaction yields were demonstrated. The carbohydrate-xanthine conjugates linked through the 1,2,3-triazole ring were evaluated as corrosion inhibitors in API 5L X70 steel, demonstrating that high inhibition efficiency has a low concentration correlation. The adsorption process of the inhibitors **3b** to **3f** follows the model of Langmuir isotherm adsorption and belongs to the physisorption-chemisorption process. As SEM and AFM results showed, the presence of an inhibitor on the metal surface decreased the corrosion process after 24 h in the corrosive solution.

## Figures and Tables

**Table 1:** Electrochemical parameters of 1-*N*-α-d-glucopyranosyl-1*H*-1,2,3-triazole xanthine as corrosion inhibitor in API 5L X70 steel immerse in HCl 1 M.

	*C* (ppm)	*R_s_* (Ω cm^2^)	*n*	*C_dl_* (µF/cm^2^)	*R_ct_* (Ω cm^2^)	*R*_mol_ (Ω cm^2^)	*R_p_* (Ω cm^2^)	*IE* (%)	χ^2^
Blank	0	5.0	0.8	7188.9	50.0	-	50.0	-	0.000452
**3** **a**	5	1.3	0.8	424.3	78.5	12.4	90.9	36.3	0.003262
	10	1.3	0.8	378.1	82.4	17.5	100.0	39.3	0.002553
	20	1.2	0.8	355.4	103.2	22.5	125.8	51.6	0.002984
	50	1.2	0.8	297.1	119.8	30.3	150.1	58.3	0.002348
**3** **b**	5	29.9	0.8	33.0	2168.4	19.3	2187.7	97.7	0.000488
	10	30.0	0.8	45.1	3617.6	13.1	3630.7	98.6	0.000217
	20	30.1	0.8	63.7	6962.0	451.1	7413.1	99.3	0.000259
	50	29.8	0.8	60.7	7200.0	425.6	7625.6	99.2	0.000238
**3** **c**	5	1.3	0.8	140.5	588.8	-	588.8	91.5	0.004295
	10	1.9	0.8	23.5	912.0	-	912.0	94.5	0.010460
	20	1.8	0.7	20.6	851.1	-	851.1	94.1	0.007602
	50	1.9	0.7	46.3	870.9	-	870.9	94.3	0.008256
**3** **d**	5	12.8	0.7	73.4	1077.4	401.7	1479.1	95.4	0.002924
	10	15.6	0.7	84.0	887.4	342.8	1230.2	94.4	0.003157
	20	24.2	0.8	15.9	3914.1	872.2	4786.3	94.3	0.001092
	50	11.2	0.8	13.0	3634.6	936.2	4570.8	94.7	0.000824
**3** **e**	5	6.6	0.8	250.5	259.6	22.1	281.8	80.7	0.000697
	10	6.7	0.8	398.1	332.6	30.3	363.0	85.0	0.000926
	20	11.9	0.7	101.8	1434.5	44.5	1479.1	96.5	0.001915
	50	10.5	0.8	117.1	465.2	366.5	831.7	89.3	0.001720
**3** **f**	5	8.5	0.4	86.2	1202.2	-	1202.2	95.8	0.001325
	10	14.3	0.6	94.9	1679.0	941.4	2620.4	97.0	0.001191
	20	16.8	0.6	102.1	1205.0	922.1	2127.1	95.9	0.001179
	50	12.5	0.5	171.3	1078.0	330.7	1408.7	95.4	0.001166

**Table 2:** Electrochemical parameters by CP at different concentrations in API 5L X70 immersed in HCl 1 M.

Inhibitor (*C* (ppm))	*E*_corr_ (mV) vs. Ag/AgCl sat	i*_corr_* (mA/cm^2^)	b_a_ (mV/dec)	b_c_ (mV/dec)	η (%)
0	−447.8	339.5	133.4	−107.2	-
**3b** (5 ppm)	−408.5	25.9	82.5	−115.9	92.4
**3b** (10 ppm)	−535.2	63.0	70.4	−144.0	81.4
**3d** (50 ppm)	−453.5	7.9	371.1	−237.7	97.7
**3e** (5 ppm)	−525.6	81.5	75.0	−144.8	76.0
**3f** (5 ppm)	−493.0	43.5	80.2	−153.7	87.2

**Table 3:** Thermodynamic analysis of carbohydrate-xanthine conjugates immersed in HCl 1 M in API 5L X70 steel rates by means of the Langmuir model.

Inhibitor	ln *K_ads_*	Δ*G*°*_ads_* (kJ mol^−1^)	Linear Regression Equation	R^2^
**3a**	8.67	−19.6	*C*/*Ɵ* = 1.6051 *C* + 0.0095	0.9999
**3b**	12.5	−28.4	*C*/*Ɵ* = 1.0021 *C* + 0.0002	0.9995
**3c**	13.2	−30.0	*C*/*Ɵ* = 1.0542 *C* + 0.0001	0.9995
**3d**	16.4	−37.3	*C*/*Ɵ* = 1.0569 *C* + 4 × 10^−6^	0.9995
**3e**	11.4	−25.9	*C*/*Ɵ* = 1.0467 *C* + 0.0006	0.9991
**3f**	14.1	−32.1	*C*/*Ɵ* = 1.0262 *C* + 4 × 10^−5^	0.9999

**Table 4:** Chemical analysis of plates observed under the microscope for the API 5L X70 steel at different conditions.

Conditions	C	O	Al	Si	S	Cl	Mn	Fe
Without inhibitor	-	44.7	-	0.0901	-	0.6275	0.8837	balance
**3b**	34.98	39.99	4.44	-	-	-	-	balance
**3f**	26.15	1.70	0.67	-	1.08	0.63	-	balance

**Table 5:** Roughness values calculated from the AFM images shown in Figure 9.

AFM Image	Ra (nm)	Rq (nm)
Polish	3.4	4.3
Without inhibitor	118.0	149
Compound **3b**	97.9	124
Compound **3f**	54.6	86

**Table 6:** B3LYP/6-311++G nucleophilicity N index, in eV, of tested molecules.

Molecule	N (eV)
**3a**	2.78
**3b**	2.87
**3c**	2.86
**3d**	2.77
**3e**	2.91
**3f**	3.12

**Table 7:** Values for EHOMO (eV) for the inhibitors in vacuum, unprotonated, fully protonated, and fully protonated in solvent.

Molecule	Unprotonated	Protonated	Protonated in Solvent
**3a**	−6.72	−11.51	−7.31
**3b**	−6.63	−11.43	−7.35
**3c**	−6.64	−11.98	−7.31
**3d**	−6.73	−11.89	−7.34
**3e**	−6.59	−10.30	−7.19
**3f**	−6.38	−11.55	−7.19

**Table 8:** ΔE (eV) values for the inhibitors in vacuum unprotonated, fully protonated, and fully protonated in solvent.

Molecule	Unprotonated	Protonated	Protonated in Solvent
**3a**	4.90	2.76	4.51
**3b**	4.99	3.01	4.67
**3c**	4.91	3.44	4.57
**3d**	5.01	3.38	4.80
**3e**	4.93	1.85	4.69
**3f**	4.93	3.63	4.80

**Table 9:** Chemical hardness (η), in eV, of unprotonated molecules, protonated forms, and in liquid phase.

Molecule	Unprotonated	Protonated	Protonated in Solvent
**3a**	2.45	1.38	2.26
**3b**	2.49	1.50	2.33
**3c**	2.46	1.72	2.29
**3d**	2.51	1.69	2.40
**3e**	2.47	0.93	2.34
**3f**	2.47	1.82	2.40

**Table 10:** Hirshfeld charges of protonated molecules.

Inhibitor Molecules											
3a		3b		3c		3d		3e		3f	
Atom	Charge	Atom	Charge	Atom	Charge	Atom	Charge	Atom	Charge	Atom	Charge
C5	0.153	C5	0.161	C5	0.147	C5	0.122	C5	0.134	C5	0.123
C4	0.054	C4	0.067	C4	0.057	C4	0.056	C4	0.058	C4	0.056
N3	0.260	N3	0.257	N3	0.252	N3	0.218	N3	0.237	N3	0.209
N2	0.047	N2	0.034	N2	0.026	N2	0.004	N2	0.018	N2	0.006
N1	0.086	N1	0.071	N1	0.090	N1	0.067	N1	0.085	N1	0.066
O10	−0.227	O8	−0.256	O10	−0.241	O8	−0.242	O10	−0.231	O8	−0.238
N9	−0.039	N6	−0.049	N9	−0.034	N6	−0.051	N9	−0.041	N6	−0.045
O12	−0.263	O7	−0.274	O12	−0.265	O7	−0.259	O12	−0.266	O7	−0.266
N11	−0.037	N9	−0.028	N11	−0.034	N9	−0.037	N11	−0.041	N9	−0.028
Oxygen atoms present in carbohydrate moieties											
O	−0.143	O	−0.178	O	−0.150	O	−0.162	O	−0.150	O	−0.143
O	−0.305	O	−0.287	O	−0.287	O	−0.219	O	−0.291	O	−0.251
O	−0.154	O	−0.170	O	−0.163	O	−0.171	O	−0.166	O	−0.152
O	−0.253	O	−0.236	O	−0.233	O	−0.279	O	−0.242	O	−0.254
O	−0.168	O	−0.163	O	−0.169	O	−0.160	O	−0.165	O	−0.193
O	−0.252	O	−0.225	O	−0.234	O	−0.233	O	−0.154	O	−0.137
O	−0.167	O	−0.174	O	−0.167	O	−0.158	O	−0.257	O	0.307
O	−0.247	O	−0.253	O	−0.304	O	−0.246	O	−0.164	O	−0.156
O	−0.194	O	−0.164	O	−0.170	O	−0.172	O	−0.253	O	−0.240
								O	−0.153	O	−0.144
								O	−0.254	O	−0.277
								O	−0.163	O	−0.136
								O	−0.264	O	−0.274
								O	−0.181	O	−0.185
								O	−0.136	O	−0.152
								O	−0.279	O	−0.252
								O	−0.179	O	−0.171

## Data Availability

Not applicable.

## Sample Availability

Samples of the compounds are available from the authors.
